# High-Throughput Analysis of Ammonia Oxidiser Community Composition via a Novel, *amoA*-Based Functional Gene Array

**DOI:** 10.1371/journal.pone.0051542

**Published:** 2012-12-19

**Authors:** Guy C. J. Abell, Stan S. Robert, Dion M. F. Frampton, John K. Volkman, Farhan Rizwi, József Csontos, Levente Bodrossy

**Affiliations:** 1 CSIRO Marine and Atmospheric Research and Wealth from Oceans National Research Flagship, Hobart, Tasmania, Australia; 2 Department of Physical Chemistry and Materials Science, Budapest University of Technology and Economics, Budapest, Hungary; Dowling College, United States of America

## Abstract

Advances in microbial ecology research are more often than not limited by the capabilities of available methodologies. Aerobic autotrophic nitrification is one of the most important and well studied microbiological processes in terrestrial and aquatic ecosystems. We have developed and validated a microbial diagnostic microarray based on the ammonia-monooxygenase subunit A (*amoA*) gene, enabling the in-depth analysis of the community structure of bacterial and archaeal ammonia oxidisers. The *amoA* microarray has been successfully applied to analyse nitrifier diversity in marine, estuarine, soil and wastewater treatment plant environments. The microarray has moderate costs for labour and consumables and enables the analysis of hundreds of environmental DNA or RNA samples per week per person. The array has been thoroughly validated with a range of individual and complex targets (*amoA* clones and environmental samples, respectively), combined with parallel analysis using traditional sequencing methods. The moderate cost and high throughput of the microarray makes it possible to adequately address broader questions of the ecology of microbial ammonia oxidation requiring high sample numbers and high resolution of the community composition.

## Introduction

The global nitrogen cycle is central to maintaining life on earth and maintaining this key geochemical process has recently been identified as one of the major threats to maintaining the Earth's environment in a habitable state [1]. Excessive nitrogen loads in coastal waters can lead to eutrophication and subsequent biodiversity loss [2], whilst retention of nitrogen in agricultural systems is key to maximising agricultural productivity.

Nitrification is an integral part of the nitrogen cycle, converting 24×10^12^ moles of N (equivalent to approximately 4×10^8^ tons of ammonia) annually on a global scale [3]. Nitrification encompasses the oxidation of ammonia to nitrite and nitrate, subsequently feeding into denitrification, which eventually leads to the formation of gaseous nitrogen forms. Nitrification is the primary process determining the rate of removal of fixed, organic nitrogen from aquatic and terrestrial ecosystems [3], [4]. From an ecological perspective, the importance of nitrification varies with the environment in which it occurs. It is an essential process for the removal of inorganic nitrogen from marine and freshwater ecosystems as well as during wastewater treatment processes, whilst in terrestrial systems, nitrification plays a major role in the removal of ammonia from soils and in doing so, increases the agricultural need for nitrogen fertilisation.

Aerobic ammonia oxidation is responsible for biological oxidation of reduced inorganic nitrogen species and is the rate limiting step in nitrification [5]. Until recently aerobic ammonia oxidation was attributed exclusively to bacterial nitrifiers (AOB) [6]. However, the recent discovery of an ammonia monooxygenase homologue associated with archaeal 16S rRNA genes during metagenomic studies [7], [8], [9] led to the discovery of archaeal ammonia oxidisers (AOA), now known to be members of the new phyla *Thaumarchaeota* [10]. A number of bacterial nitrifiers have been isolated in pure culture and shown to belong to both the gamma- and beta-*Proteobacteria* with different groups associated with different environments [11]. Whilst traditional cultivation methods have, so far, have had limited success in isolating AOA, a number have been enriched and subsequently described [12], [13], [14], [15], [16]. All of these have been shown to be capable of stoichiometric conversion of ammonia to nitrite and some appear to have high affinities for ammonia suggesting that they are capable of nitrification at lower ammonia concentrations than cultivated AOB.

Previous studies have demonstrated that environments dominated by AOA include soils [17], open ocean waters (where their abundance is as high as 20% of all bacteria and archaea [18]), and some estuaries [19], [20]. Conversely AOB have been shown to dominate in estuarine sediments [21], wastewater treatment plants [22], [23] and zinc-contaminated soil [24].

The reason for the presence of both seemingly functionally equivalent groups in the environment is unclear, although it has been suggested that pH [25], nitrite concentrations [26], sulphide and phosphate concentrations [27] and salinity [19] may be important factors, whilst salinity, oxygen and hydrological factors may shape the structure of these communities [20], [21], [28], [29], [30], [31].

Recent genomic and metagenomic studies raised the possibility that members of the AOA may be capable of mixotrophic metabolism [32]. As such, it cannot be excluded that members of the AOA and also AOB represented by divergent ammonia monooxygenase (*amoA*) phylotypes may be capable of utilising compounds such as acetate or methane [33]. Whilst many of these organisms are not currently cultivated, studying their occurrence in different environments may help to describe their ecology. Such community scale ecology approaches, applied across large numbers of samples and which relate abundance, environment and physical variables may be the best approaches to elucidating the ecology and even physiology of these, mostly, uncultivated organisms.

To overcome the limitation associated with cultivation, molecular methods have been broadly used to assess the diversity of nitrifiers in various environments. These methods have allowed nitrifiers to be quantified and characterized by analysis of functional marker genes involved in the nitrification process. The gene encoding the alpha subunit of ammonia monooxygenase (*amoA*) is a widely used marker for molecular studies of ammonia oxidising microorganisms in environmental systems. Similar evolutionary relationships based on *amoA* and 16S phylogeny have been shown previously, supporting the use as of *amoA* as a phylogenetic marker [6], whilst groups of similar amoA phylogenies have been shown to have similar ammonia oxidising physiologies [34]. The importance of *amoA* as a molecular marker for ammonia oxidisers is highlighted by a search of ISI Web of Knowledge, that revealed 622 publications involving the use of *amoA* as a molecular marker, with 584 subsequent to the first paper using it at a community scale [35] and 120 publications in 2011 alone. A number of different molecular methods have been used to assess the diversity of environmental *amoA* genes including DGGE [36], [37] as well as sequencing approaches [6], [38]. A recent study [39] identified TRFLP analysis of ammonia oxidiser community structure as well as functional gene array technology as key biological indicators for the assessment of soil function.

Microarrays are tools for the highly parallel hybridisation of a single target to a multitude of probes. Microbial diagnostic microarrays (MDMs) contain hundreds to tens of thousands of nucleic acid probes, each specific to a given microbial taxon (strain, subspecies, species, genus or higher phylum). The most common MDMs are phylochips and functional gene arrays (FGAs). Phylochips are typically based on the broadly used phylogenetic marker, the 16S rRNA gene [40], [41], [42], [43]. FGAs are based on functional genes and their probe sets reflect the phylogeny of the functional gene(s) targeted [4], [44], [45], [46], [47], [48]. If the phylogenies of the functional gene and of the microbes carrying them are reasonably concordant, an FGA can deliver phylogenetically relevant information focused on a functional clade [6], [49], [50].

Here we report on the development and thorough validation of an *amoA*-based short oligonucleotide microarray and associated methodology for the affordable, fast, high-throughput, in-depth analysis of the community structure of aerobic autotrophic ammonia oxidising bacteria and archaea from a wide range (marine, estuarine, soil and wastewater treatment plant) of environments.

## Results

### Method development

The *amoA* microarray is based on individually selected, short oligonucleotide (18–28 mer) probes and uses PCR amplified, Cy3-labelled targets for hybridisation. The method used for the *amoA* array is based on those used for a previously published and widely applied *pmoA* array for methanotrophs [46], [51], [52]. Details on the development and optimisation of the method are published in [53].

The development of a new microarray provided us with opportunities to further improve the methods used, focusing on cost effectiveness and therefore affordability. A triple chamber custom-designed Hybriwell has been applied, significantly reducing hands-on time for microarray spotting, slide processing, hybridisation and scanning. Reaction volumes for both labelling and hybridisation have been lowered, reducing consumables costs. Finally, a magnetic bead based PCR purification method has been adapted, minimising nucleic acid purification time and expense. A further advantage of using this method is that it is suitable for use with liquid handling robots.

### Microarray design

**Figure 1:**
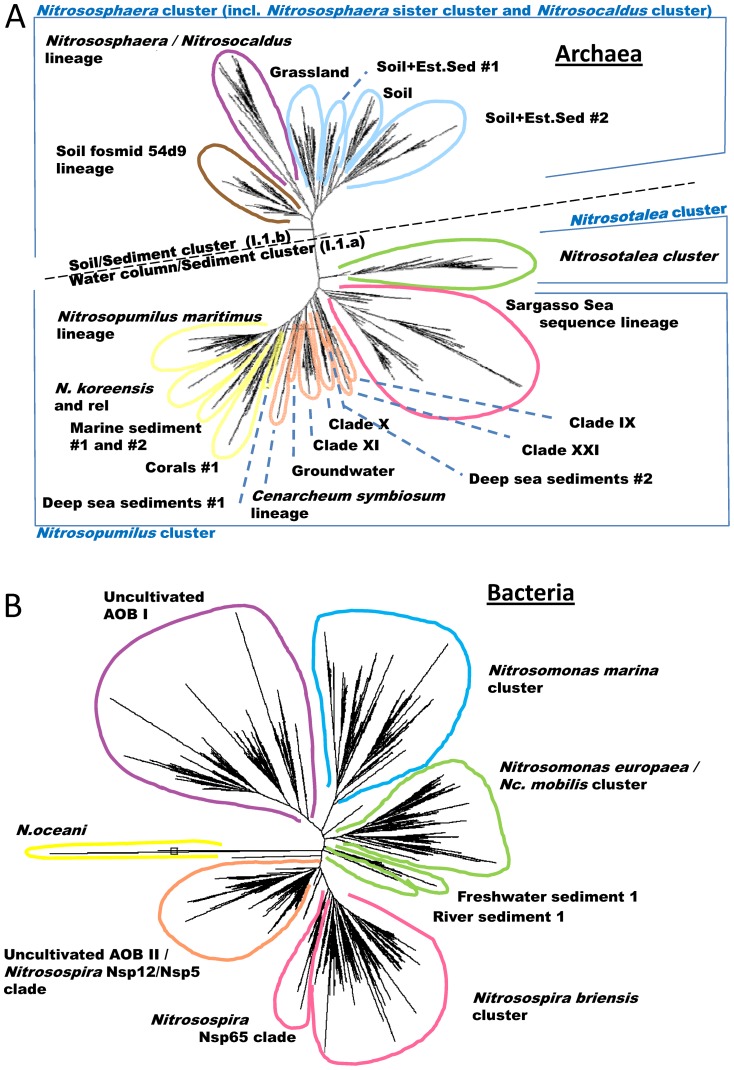
Radial phylogenetic trees of A) archaeal and B) bacterial *amoA* sequences. High level clades are indicated by different colours, which are also used in supplementary information (Supporting Information S7, evaluation with environmental samples) where appropriate. Note: the colours indicating high level clades on this figure do not correspond to the colours used on Supporting Information S1 (probe set specificities). On the AOA tree, clusters from a very recent review on AOA phylogeny are also shown in blue [38]. For the exact position of the *Nitrososphaera* sister cluster and *Nitrosocaldus* cluster, please refer to Supporting Information S1. Soil+Est.Sed = Soil+Estuarine Sediment.

We created a comprehensive database based of all the publicly available bacterial and archaeal *amoA* sequences and extended it with previously unpublished sequences (arising from our own work as well as courtesy of collaborators, see Acknowledgements). Phylogenetic trees were created including all acceptable length and quality sequences (>20,000 sequences in total) and used to guide the probe design (Fig. 1 and Supporting Information S1). An ARB database consisting of 838 sequences, including a number of representative sequences for each clade and with phylogenetic trees matching the topology and naming convention in the figures is available from the authors upon request. Probe design followed the principles applied for the development of a *pmoA*-based microarray for methane oxidisers [46] and followed the multiple-probe concept [41]. We designed 354 probes, including positive controls targeting the broadly used *amoA* PCR primers and an external spike control (Supporting Information S2). Probes were designed to target the core alignment positions 1124–1616 for the AOA and 1320–1771 for the AOB (for details see Supporting Information S3 - an ARB database with representative sequences from all clades). These regions correspond to the majority of the published PCR primers (Table 1). The use of alternative *amoA* PCR primers [54] that fall within this alignment core may exclude some of the regions targeted by the probe set and, as such, may not be suitable for use with the array (for details see Supporting Information S4).

**Table 1:** PCR primers and conditions.

PCR primer	Specificity	Sequence (5′-3′)	L	dir	T_ann_	#	Ref
amoA-1F	β-Proteo-bacterial *amoA*	GGGGTTTCTACTGGTGGT	18	fw	53	35	[35]
T7-amoA-2R		TAATACGACTCACTATAG CCCCTCKGSAAAGCCTTCTTC	21	rev			
AOA111F	Archaeal *amoA*	TTYTAYACHGAYTGGGCHTGGACATC	26	fw	53	35	[8]
T7-AOA643R		TAATACGACTCACTATAG TCCCACTTWGACCARGCGGCCATCCA	26	rev			
Arch-amoAF	Archaeal *amoA*	STAATGGTCTGGCTTAGACG	20	fw	53	35	[70]
T7-Arch-amoAR		TAATACGACTCACTATAGGCGGCCATCCATCTGTATGT	20	rev			

L = length. dir = direction. fw = forward. rev = reverse. T_ann_ = annealing temperature. # = number of PCR cycles used. Ref = reference.

### Array validation

**Figure 2:**
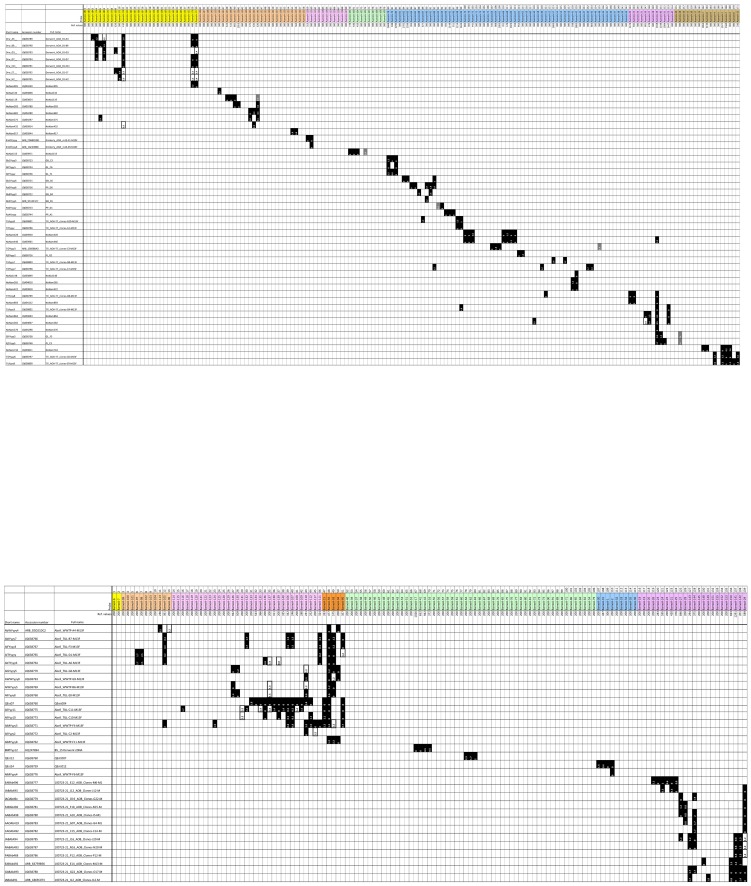
Validation with pure reference targets. Range of strain coverage for the oligonucleotide probe set targeting *amoA* genes of AOAs (Fig. 2A) and AOBs (Fig. 2B). A similar table in which over 20,000 sequences were considered (without hybridisation results) is shown in Supporting Information S6. Black fill indicates expected positive results, grey fill indicates positive results not predicted and thick black framing indicates negative results where hybridisation was predicted. Numbers indicate the number of weighted mismatches as described in the relevant section of Experimental Procedures. Reference signal values (% of that of positive controls) obtained with full match targets are indicated (‘Ref. values’). NOTE: unpredicted positives left are either from broad specificity probes where signal is still preferred or from probes not yet validated with a perfect match reference target where the signal intensity for positive call is undetermined (i.e., probes with higher than average binding capacities). Details are readable once the figure is magnified to A3 size.

A set of 81 reference targets consisting of pure *amoA* clones was used to validate the microarray (Fig. 2). The panel of reference targets covered the majority of the known *amoA* sequence diversity, with special emphasis on having a perfect match or <0.5 wMM (weighted mismatches) target against probes on the microarray wherever possible. Probe-target combinations with less than 1.5 wMM were expected to yield a signal above cut-off. Probe-target combinations with wMM values above 2.5 were expected to yield no positive signal. Finally, wMM values between 1.5 and 2.5 were considered potentially positive; however probe specificities were re-evaluated taking into consideration the results of validation with reference targets.

After validation of the probe set, 13584 out of 13656 individual hybridisation reactions (184 and 145 probes hybridised with 49 and 32 targets for AOA and AOB, respectively) gave results that matched the expected probe specificities (99.5% correct results). One hundred and ten probes were validated with a perfect match target; of these 110 probes 13 were removed from the final probe set due to false positive results. Supporting Information S5 contains two tables displaying the wMM values for each probe-target pair for the bacterial and archaeal *amoA* probe sets, respectively. Supporting Information S6 is a shortened version of the same tables, based on a small, representative subset of the *pmoA* sequence database. Supporting Information S1 shows the expected probe specificities considering original probe design criteria as well as subsequent validation results. No hybridisation was observed by AOA targets on AOB probes and vice versa (data not shown).

Samples from a variety of environments were used to test the performance of the microarray with natural microbial assemblages (Supporting Information S7). Samples included estuarine sediment, agricultural soil, mixed liquor from wastewater treatment plants and biomass filtered from marine water. Microarray hybridisation results were compared to clone libraries generated from the same samples. Clone libraries generated from *amoA* PCR products contained between 73 and 332 *amoA* clones. Comparison of the microarray and clone library results indicated a high degree of consistency across the different environmental samples analysed (Table 2). The extensive evaluation with environmental samples (see below) resulted in only 5 cases where a positive probe was not associated with a corresponding sequence in the clone library. There was a single case where a strong positive signal on the microarray (probe AamoA-159) was not associated with corresponding sequences detected; most likely indicating a false positive hybridisation not yet detected by validation with single reference targets. While none of the reference targets obtained from the same environmental samples (Derwent estuary, Tasmania) resulted in the same false positive signal, the probe has been considered potentially false positive and thus removed from the probe set. There were no sequences found in the clone libraries that did not elicit expected positive probe responses.

**Table 2:** Summary of evaluation with environmental samples.

			Clades			Probes					
Environment	No. of samples tested	Organisms tested	Clades detected by array	Clades detected by clone library	Δ	Positive probes	Supported by clone library	Δ	Δ %	Library size	Comment
Agricultural soil	3	AOB	5	5	0	23	22	1	4.3%	82	Weak signal on array – potentially missed by small clone library
Temperate estuary, sediment	6	AOA	3	3	0	10*	10	0	0.0%	81	*Probe AamoA-159 has been removed from the probe set due to a strong positive signal with these samples. See text for details.
Temperate estuary, sediment	6	AOB	6	6	0	13	12	1	7.7%	332	Moderate signal on array+binding capacity not known – potentially missed by moderate size clone library; Sequences from this clade previously found in the same environment (Abell, unpublished)
Wastewater treatment plant	3	AOB	6	6	0	16	15	1	6.3%	83	Weak signal on array – potentially missed by small clone library
Open ocean	4	AOA	3	2	1	7	6	1	14.3%	73	Weak signal on array – potentially missed by small clone library

Δ = difference between array and clone library results.

Analysis of the ammonia oxidising community in an agricultural soil using the AOB array demonstrated strong signals for universal probes targeting the *Nitrosospira* lineage (probes BamoA-15, BamoA-92, BamoA-93, BamoA-94, BamoA-95 and BamoA-111) as well as higher level probes targeting the *N. briensis* group (probes BamoA-106, BamoA-107, BamoA-109, BamoA-110 and BamoA-112) across all three samples. Specific probes that gave positive signals indicated the presence of NSP65 clade (BamoA-97 and BamoA-105); clade 321 (BamoA-116); *N. briensis* clades 287, 288, 216 and 330 (BamoA-126, BamoA-130 and BamoA-132) as well as NSP2 and NSP17 related clades (BamoA-134, BamoA-135, BamoA-136, BamoA-138, BamoA-139 and BamoA-140) (Supporting Information S7).

**Figure 3:**
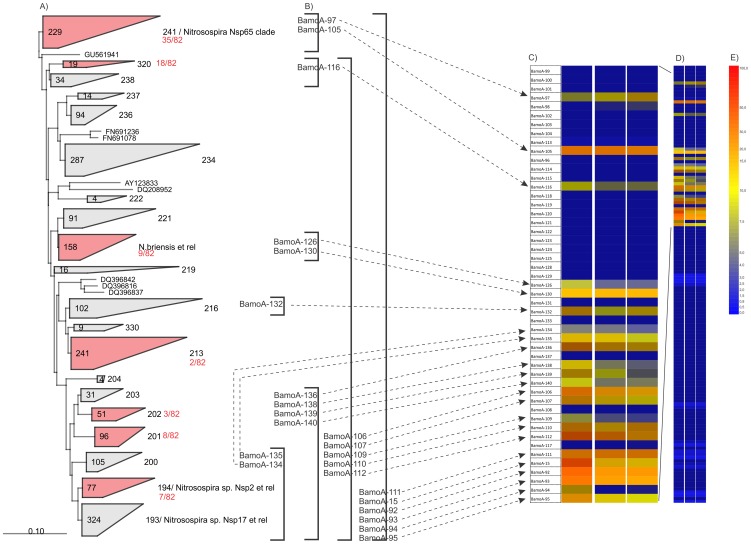
Evaluation with environmental samples – Agricultural soil example. A) Phylogenetic tree showing clades detected by clone library sequencing. Numbers within boxes representing clades indicate the number of sequences comprising clades. B) Coverage of probes found positive. C) Microarray results; only the section with positive results shown. D) Full microarray results. E) Side bar indicating colour coding (red: maximum signal, 100%; blue: no signal, 0%).

A clone library of 96 clones resulted in 82 *amoA* clones, belonging to clades Nsp65, 320, *N. briensis et rel.*, 213, 201, 202 and Nsp2 *et rel.* (Fig. 3). This clone library explains every positive signal seen on the microarray with the exception of BamoA-132 (Fig. 3).

Analysis of estuarine sediment samples from a temperate estuary was performed using both the AOA and AOB arrays. Analysis using the AOB array demonstrated positive signals only for probes corresponding to the uncultivated AOB I group. Probes BamoA-141, BamoA-142 and BamoA-29, corresponding to the whole group, gave positive signals in all or most samples. Probe BamoA-150, targeting the subcluster BamoA-127 within the Uncultivated AOB 1 group was also positive in most samples and supported the probes BamoA-143 and BamoA-144 (targeting cluster 85) as well as probes BamoA-145 and BamoA-148 (targeting clusters 76 and 69 respectively). Probes BamoA-27 and BamoA-28 gave positive signals in some samples indicating the presence of sequences corresponding to Monterey Bay ‘A’ group, whilst probes BamoA-25, BamoA-21, BamoA-24 and BamoA-23 gave positive signals in sample 1 (mouth of the estuary) indicating the presence of group 113 in this sample (Supporting Information S7). A clone library of 384 clones resulted in 332 *amoA* sequences, belonging to clades 69, 85, 90, 105, 107, 113, 116 and 121 (data not shown). This clone library explains every signal seen on the microarray with the exception of BamoA-145.

Analysis using the AOA array demonstrated positive signals for probes corresponding to the *Nitrosopumilus maritimus* and ‘Marine sediment’ lineages only. Probes AamoA-176, AamoA-177 and AamoA-178, targeting these two groups plus the *N. koreensis* cluster were positive in all 6 samples. Probes AamoA-174, AamoA-180 and AamoA-181, targeting the *N. maritimus* lineage were also positive in all 6 samples, whilst there were weak hybridisation signals for probes 183 and 184 targeting subgroups within this cluster. Probes targeting the Marine sediment #1 cluster (AamoA-169 and AamoA-171) were strongly positive in 1–4, but weak or absent in samples 5 and 6 (upstream samples). Probe AamoA-159 gave a positive signal in all samples but corresponding higher level probes were negative so this probe was removed from the array as a likely false positive (Supporting Information S7). A clone library of 96 clones resulted in 82 *amoA* clones, belonging to clades 165, 167, 168, 170, *N. pumilus et rel.*, 179, 180 within the *N. maritimus lineage* and 153 and 154 within the Marine sediment #1 (data not shown). This clone library explains every signal seen on the microarray with the exception of AamoA-159 (mentioned above).

Analysis of the AOB communities within a wastewater treatment plant (WWTP) indicated the presence of sequences related to *Nitrosomonas eutropha* (probes BamoA-30, BamoA-31, BamoA-1 and BamoA-2) as well as members of the *Nitrosospira* lineage (probes BamoA-92, BamoA-93 and BamoA-94). Specific probes targeting the broader *N. briensis* cluster (BamoA-110, BamoA-111 and BamoA-112) as well as more specific probes targeting the *N. briensis* clades 287 and 288 (BamoA-126) as well as the clades 272, Nsp12/Nsp5 and Nsp65 within the *Nitrosospira* Nsp12/Nsp5 cluster (BamoA-97, BamoA-113 and BamoA-105) gave positive signals (Supporting Information S7). A clone library of 96 clones resulted in 83 *amoA* clones, belonging to clades 298 (within *Nitrosomonas eutropha*) and 272, 273, 274, 241 (data not shown). This clone library explains every signal seen on the microarray with the exception of BamoA-33.

AOA communities in samples from filtered open ocean and coastal seawater were analysed using the AOA array which demonstrated the presence of the Sargasso Sea sequence group ‘Water column A’ (probes AamoA-110 and AamoA-111) in all samples. Probes targeting the *Nitrosopumilus maritimus* lineage (AamoA-180, AamoA-178 and AamoA-176) were positive in the two coastal samples while negative in the two open ocean samples. Probe AamoA-144 was positive in 3 out of 4 marine samples, indicating the presence of a clade so far only detected in freshwater environments [55], [56] (Supporting Information S7). A clone library of 96 clones resulted in 73 *amoA* clones, belonging to clades *N. maritimus et rel*. within the *N. maritimus* lineage and to 109 and 110 within the ‘Water column A’ cluster (data not shown). This clone library explains every signal seen on the microarray with the exception of AamoA-144.

### Array performance

The *amoA* microarray is based on short oligonucleotide probes and PCR amplified target. The short oligoprobes on the array, in line with earlier findings, allowed the reliable discrimination of 2 bp mismatches. This, in turn, enabled the design of probes with high phylogenetic resolution with probes differentiating clades with less, than 7% sequence dissimilarities. The detection limit is approximately 5% of the population analysed [50]. The consumables costs for running an analysis with the microarray at the time of print are approximately 11 USD (starting from purified environmental DNA). One researcher can routinely analyse 40 samples within 24 hours (including overnight hybridisation).

## Discussion

We have developed a functional gene microarray, based on the *amoA* gene, for the characterisation of the ammonia oxidising microbial community from environmental samples. The microarray and the associated methodology provide high resolution community composition analysis at high throughput and affordable cost. The microarray resolves most of the ammonia oxidising community to the species level (87% and 80% *amoA* similarity levels for AOA and AOB, respectively [6], [38]) and below. The array technique proved to be robust in this and another study [57]. Array results agreed with conventional clone library analyses and in both studies the array detected several groups not found by sequence analysis. The use of next generation sequencing is likely to provide equivalent or higher sensitivity to the microarray technique, however at increased cost. Consumable costs of the analysis with this microarray are approximately 10 times lower than typical next generation (tagged) sequencing analyses offered by commercial suppliers. Given that most of the analysis is inherently integrated into the microarray technique, the time required for analysing results is also substantially lower than that for next generation sequencing data [58]. Both consumable costs and labour are roughly the same as for widely used fingerprinting techniques, like tRFLP, ARISA or DGGE. However, the microarray provides additional information by also identifying the organisms present. To the best knowledge of the authors, there are three environmental microarrays targeting fully or partly the *amoA* gene [4], [47], [48]. All of these arrays are based on long oligonucleotide probes which in turn limit the phylogenetic resolution of the assay [51].

Quantification of nitrifying bacteria and archaea based on *amoA* sequences is potentially biased by a variation in the number of *amoA* gene copies per genome (1–3 in most cases) [59]. This has to be taken into account when interpreting *amoA* based molecular fingerprinting results, whether using tRFLP, next generation sequencing or microarrays.

Validation of the microarray with pure (PCR amplified) reference targets demonstrated a good success rate of probe design (99.5% correct hybridisation results; 88% of the probes validated behaved as predicted, in all of the hybridisation reactions). The success of probe validation, combined with the use of multiple-probes targeting individual groups increases the confidence in the detection of specific groups using the microarray method. Further support for the specificity of the array is provided by the evaluation of parallel clone libraries and arrays from a number of environmental samples. As the array is applied to new environments, creation of parallel reference clone libraries allow further validation and updating of the array [46], [58], [60] thereby ensuring proper coverage and specificity of the probe set for all target environments.

During validation with complex environmental samples, five probes gave signals that were not accounted for in the corresponding clone libraries. Four of these probes (BamoA-145 with estuarine sediment; BamoA-132 with soil; BamoA-33 with WWTP sample; AamoA-144 with marine water) (Supporting Information S7) gave only weak signals and as such are likely to target sequences that were in low abundance in the sample, and therefore were not accounted for by the clone libraries.

Probe BamoA-145 which was positive with estuarine sediment samples targets clade 78 within Uncultivated AOB I (Supporting Information S1), which is entirely comprised of sequences from estuarine and salt marsh sediments [21], [30], [61]. The presence of this probe was also supported by positive signals in corresponding probes with broader specificity (probes BamoA-141, BamoA-142, BamoA-29; see also Supporting Information S1).

Probe BamoA-132 gave a positive signal during the analysis of soil samples (Fig. 3). This probe targets clades 216 and 330 within the *N. briensis* cluster (Supporting Information S1). Both of these clades contain sequences exclusively found in soils [62], [63]. The positive signal from this probe was also supported by corresponding probes with broader specificities (see Fig. 3).

Probe BamoA-33 which gave a positive signal in the WWTP samples targets *N. communis/N. nitrosa et rel*. and clade 34 within the *Nitrosomonas europaea*/*Nc. mobilis* cluster. Sequences within these groups have previously been detected in WWTPs [64], [65]. Unfortunately it was not possible to design a broader level probe for these groups and the more specific probe corresponding to a subset of these groups (BamoA-32) gave no signal. It is therefore not possible to rule out this signal as being a false positive.

Probe AamoA-144, detected in the marine water samples targets the ‘Groundwater’ clade (Supporting Information S7). This group exclusively contains sequences from freshwater environments and therefore, in the absence of corresponding broader level probes it is not possible to rule out this representing a false positive signal.

Previous estimates of a similar array methodology indicate that the sensitivity of the array is approximately 5% [46]. In the present study analysis of five different environmental samples with the *amoA* array indicated only a single probe (AamoA-159, Supporting Information S7) displaying false positive results with no corresponding target sequence detected by clone libraries (data not shown).

The analysis of a number of different environmental samples using the array demonstrates its suitability for analysing any environmental sample from which *amoA* can be amplified. A total of 12 and 10 environmental samples were analysed with the AOB and AOA arrays respectively (Supporting Information S7). The analysis involved approximately 6 hours of total hands-on time and 250 AUD consumables cost and resulted in a high resolution picture of the ammonia oxidising microbial communities from these 22 samples. The analysis of a number of samples from each of the different environments demonstrates the potential of the array for analysing the differences within and between individual environments, providing a rapid and detailed assessment of the alpha and beta diversity of ammonia oxidising bacteria and archaea. A detailed description of each of the environments discussed here is beyond the scope of this paper and will be comprehensively elucidated in subsequent studies. In addition, and as demonstrated previously with a methanotroph array, the methodology can be easily adapted to analyse environmental RNA [66], [67]. This approach provides information indicating the activity of the various members of the microbial community rather than presence only.

In summary, we have developed a new tool - a short oligonucleotide based microarray for the high resolution analysis of the community composition of ammonia oxidising bacteria and archaea from a wide range of environments. The moderate cost and labour requirements of the methodology allows for the analysis of hundreds of environmental DNA or RNA samples per week by a single investigator with a modest budget. This throughput in turn makes it possible to address broader fundamental and applied questions of the ecology of microbial ammonia oxidation, i.e., community structure-function relationships, ecological stability, spatial and temporal dynamics, etc. The ongoing and iterative probe validation/design process will allow the *amoA* microarray to be further refined and kept up to date with the discovery of novel *amoA* sequences.

## Experimental Procedures

### Environmental samples

Environmental samples used for the validation of the microarray were: i) 6 estuarine sediment samples from the Derwent river, Tasmania, Australia (n = 6); ii) 3 agricultural soil samples from Harden, New South Wales, Australia (n = 3); iii) 3 activated sludge samples from a wastewater treatment plant in Sydney, New South Wales, Australia (n = 3); and iv) 4 open ocean water samples from the Kimberley region, Western Australia, Australia (n = 4) (Table 2). The aim of using these samples was solely to demonstrate the applicability of the microarray to a variety of environments. These environments are subject to separate, more detailed studies and their detailed characterisation will be published.

### Microarray design

Database and phylogenetic trees were constructed and oligonucleotide probes were designed using the phylogenetic software package ARB [68]. A comprehensive database containing all published bacterial and archaeal *amoA* sequences, as well as many unpublished sequences was established. Alignments were made using the integrated aligner function in ARB_EDIT. Phylogenetic trees, constructed from nearly full length sequences using the ARB Neighbour Joining function and updated with partial sequences using the ARB Parsimony function were used to guide the probe design process. The size of the sequence dataset made it practically impossible to calculate maximum likelihood phylogenies. The trees published in this paper are meant to illustrate probe specificities, and may slightly differ in deep branching patterns from phylogenetic trees calculated by different methods. Probes were designed using the Probe Design and Probe Match functions, accessing a PT-server database created from the above ARB database. A custom program, Batch Probe, was developed for the fast, automated generation of all Probe Match outputs, starting from an input list of probe names and sequences in a text file format. The Batch Probe program provides a script called ‘arb_probe’ that takes in as arguments serverid, number of match mismatches and the match sequence, returning the list of matches with some header information to stdout. For this work a perl script was written that takes in as a single argument the name of an ascii file containing a list of filenames and sequences to match that are comma separated. It then calls arb_probe in a loop with each of the sequences provided and uses regular expressions to format the returned text so as to only write the matching sequences, in rows, to the filename provided for that sequence. In this instance the serverid (0) and number of match mismatches (3) were hard-coded in the script. The Batch Probe program and instructions for use are available from the authors upon request. Probe Match outputs were imported into CalcOligo 2.03 (www.CalcOligo.info). CalcOligo was used to create an Excel table indicating predicted melting temperatures (based on the nearest neighbor model and SantaLucia parameters), length and GC content of the probes and the number of weighted mismatches between each probe-target pair. Nearest neighbor T_m_ values were calculated with concentration settings of 250 nmol for oligonucleotide and 50 mmol for Na^+^. Factors for weighing mismatches in CalcOligo were as follows. Positions: 5′ 1^st^ 0.3; 5′ 2^nd^ 0.6; 5′ 3^rd^ 1.0; 3′ 1^st^ 0.3; 3′ 2^nd^ 0.8; 3′ 3^rd^ 1.1; all other positions 1.2. Basepairs: dArC 1.2; dTrC 1.2; dGrU 0.7; dTrG 0.4; all other mismatched basepairs 1.0. Probe-target pairs with weighted mismatch values of up to 1.5 were expected to yield positive hybridisation under the conditions applied. A detailed description of the probe design process has been published elsewhere [53].

### Microarray preparation

Oligonucleotides for immobilization were custom synthesized (Integrated DNA Technologies, Coralville, Iowa, USA) with a 5′ NH_2_ group, followed by a C_6_ spacer and five thymidine residues preceding the probe sequence [46]. A 384 well flat bottom plate was prepared with 30 µl of 50 µM oligonucleotide solutions in 50% DMSO. Samples were spotted with a NanoPrint spotter, using a single 946MP3 pin (ArrayIt Corp., Sunnyvale, CA, USA) at 55 % relative humidity at room temperature onto VSS aldehyde slides (Cel Associates, Houston, USA). Arrays were always spotted in triplicate to enable a statistical correction for errors (Supporting Information S8). Spotted slides were incubated overnight at room temperature at <30% relative humidity, rinsed twice in 0.2% (w/v) SDS for 2 min at room temperature with vigorous agitation to remove the unbound DNA. Slides were then rinsed twice in distilled water (dH_2_O) for 2 min at room temperature with vigorous agitation, transferred into dH_2_O, preheated to 95–100°C for 2 min, and allowed to cool at room temperature (∼5 min). Slides were treated in a freshly (immediately before use) prepared sodium borohydride solution for 5 min at room temperature to reduce free aldehydes. Preparation of the sodium borohydride solution: 0.5 g NaBH4 was dissolved in 150 ml phosphate-buffered saline (PBS; 8 g NaCl, 0.2 g KCl, 1.44 g Na_2_HPO_4_, 0.24 g KH_2_PO_4_, in 1000 ml H_2_O, pH 7.4, autoclaved) then 44 ml of 100% ethanol was added to reduce bubbling. Slides were rinsed three times in 0.2% (w/v) SDS and once in dH_2_O for 1 min each at room temperature. Finally, slides were dried individually using an airgun fitted with a cotton wool filter (to keep oil microdroplets away from the slide surface). Dried slides were stored at room temperature and at low humidity in the dark prior to use.

The quality of the spotted arrays was checked by scanning every slide in the 532 nm channel after spotting to ensure that each probe has been spotted. In addition, the first slide of each print batch was hybridised with a known mixture of pure PCR products to check the homogeneity of the spots and the performance of the glass substrate.

### Environmental DNA purification

Soil and sediment samples (∼10 g) were homogenised and a 0.5 g subsample was used for DNA extraction. One ml of homogenised activated sludge was centrifuged at 10000× g for 5 minutes and the pellet used for DNA extraction. Water samples (1 L) were filtered onto 0.2 µm filters as previously described [69] and the whole filter used for DNA extraction. All environmental samples were frozen immediately after sampling and subsequently stored at −20°C until processing.

Following subsampling and pre-processing all DNA extractions were performed using a bead beating method described previously [20].

### Target preparation

*amoA* genes were amplified using the primers listed in Table 1. The reverse primers contained the T7 promoter site (5′-TAATACGACTCACTATAG-3′) at their 5′ end, which enabled T7 RNA polymerase mediated *in vitro* transcription using the PCR products as templates. For each target, three PCR reactions of 50 µl volume each, consisting of 1× FailSafe Premix G buffer (Epicentre Biotechnologies, Madison, WI, USA), 20 pmoles of both primers, 10–30 ng environmental DNA or 0.1 ng cloned PCR product as template, and 1 U of Taq polymerase (Invitrogen), were performed in an Eppendorf DNA thermal cycler (Eppendorf, Hamburg, Germany) using amplification conditions as listed in Table 1. PCR products were pooled and purified using the Agencourt AMPure XP PCR purification kit (Beckman Coulter, Danver, MA, USA), according to manufacturer's instructions. Purified DNA was dissolved in ultrapure water to a DNA concentration of 50 ng/µl and stored at −20°C. Working under RNAse-free conditions, *in vitro* transcription was carried out as follows: 2.8 µl purified PCR product (50 ng/µl), 1.6 µl 5× T7 RNA polymerase buffer, 0.8 µl DTT (100 mM), 0.2 µl RiboSafe RNAse inhibitor (40 U/µl) (Bioline), 0.4 µl each of ATP, CTP, GTP (10 mM), 0.2 µl UTP (10 mM), 0.4 µl T7 RNA polymerase (40 U/µl) (Invitrogen) and 0.2 µl cyanine3-UTP (10 mM) (Perkin Elmer) were added into PCR tubes or wells in PCR plates and incubated at 37°C for 4 hours in a PCR thermocycler with heated lid function. RNA was purified immediately using the Agencourt RNAClean XP kit (Beckman Coulter, Denver, MA, USA), according to manufacturer's instructions. Purified RNA was eluted into 40 µl dH_2_O. Purified RNA was fragmented by incubating with 10 mM ZnCl_2_ and 25 mM Tris.Cl (pH 7.4) at 60°C for 30 min. Fragmentation was stopped by the addition of 10 mM EDTA pH 8.0 to the reaction and placing the reaction on ice. RiboSafe RNAse inhibitor (1 µl, 40 U/µl, Bioline) was added to the fragmented target. Fragmented, labeled RNA targets were stored at −20°C.

### Hybridisation

No prehybridisation was done. A rotary hybridisation oven and conventional hybridisation tubes were preheated for at least 2 hours at 60°C. HybriWell (Grace BioLabs) stick-on hybridisation chambers (custom made, containing 3 chambers per slide, 100 µl each in volume, order number 46170) were applied onto the slides containing the arrays. Assembled slides were preheated on top of a dry heating block preheated to 60°C. For each hybridisation, 57 µl DEPC-treated water, 1 µl 10% SDS, 2 µl 50× Denhardt's reagent (Sigma), 30 µl 20× SSC and 10 µl target RNA were added to a 1.5 ml Eppendorf tube and incubated at 65°C for 1–15 min. Preheated hybridisation mixtures were applied onto assembled slides, chambers were sealed with seal spots (Grace BioLabs). Slides were placed into preheated, conventional hybridisation tubes and incubated overnight at 60°C in a rotary hybridisation oven at lowest rotation setting (approximately 10 rpm).Following hybridisation, HybriWell chambers were individually removed and slides were immersed immediately into 2×SSC, 0.1% (w/v) SDS at room temperature. Slides were washed by shaking at room temperature for 5 min in 2×SSC, 0.1% (w/v) SDS; twice for 5 min in 0.2× SSC and finally for 5 min in 0.1× SSC. Slides were dried individually using an airgun with an internal cotton wool filter. Slides were stored at room temperature in the dark and scanned the same day.

### Scanning and data analysis

Hybridised slides were scanned at 10 µm resolution with a GenePix 4000B laser scanner (Molecular Devices, Sunnyvale, CA, USA) at wavelengths of 532 nm and 635 nm for Cy3 and Cy5, respectively. Fluorescent images were captured as multi-layer tiff images and analysed with the GenePix Pro 6.0 software (Molecular Devices). Hybridisation signal (median of signal minus background) for each probe was expressed as percentage of the average signal of the positive control probes on the same array. Positive controls used were: AOA11F, AOA643R, Arch-amoAF and Arch-amoAR for the archaeal and amoA-2Rc (only) for the bacterial array. As each microarray consisted of triplicate subarrays, normalized signal intensities of the triplicate spots within an array were used to determine average results and standard deviations. Several probes produced non-specific background signal up to 3% of their maximum signal (obtained with perfect match targets).Hybridisation between a probe and a target was thus considered positive if the signal was at least 5% of the strongest signal obtained for that probe with the validation set of reference strains/clones. For probes, where no perfect match reference target was available or the strongest signal was less than 300 or 200 (% of the signal obtained on the positive controls) for the archaeal and bacterial array, respectively, this reference value was arbitrarily set to 300 or 200 (for the archaeal and bacterial arrays, respectively). This was found to minimize false positive calls while not creating any false negative call. While no dedicated negative controls were applied, for each individual hybridisation over 70% of all probes present on the array functioned effectively as negative controls for each individual hybridisation.

### Evaluation with environmental samples

*amoA* PCR amplicons obtained from environmental samples were cloned and sequenced as described previously [20]. Phylogenetic analysis of *amoA* sequences arising from this study was performed using the ARB software package [68]. The initial ARB database and phylogenetic trees used for probe design were updated with the new sequences and weighted mismatch tables were calculated using CalcOligo following the same approach as described under ‘Oligonucleotide probe design’. Microarray hybridisation data were compared to the expected signals, based on the clone libraries and predicted probe specificities.

Results of individual microarray experiments were first normalized to positive control probes, and then to the reference values determined individually for each probe, averaged between replicate spots and displayed as a heatmap, using the GeneSpring software. In essence, a value of 100% (red) indicates maximum achievable signal for an individual probe, whereas a value of 10% (yellow) indicates that about 10% of the total PCR product hybridised to that probe.

### Nucleotide sequence accession numbers

Accession numbers for the partial *amoA* sequences used to validate the probe set, including those obtained in this study, are shown in Fig. 2.

## Supporting Information

Supporting Information S1 **Phylogentic tree with probe specificities (multiple probe concept).** Coloured boxes indicate the specificity of the probe on the phylogenetic tree. Corresponding probe names are displayed to the right in the same colour (‘Probe name’). Boxes with dotted lines indicate probes with partial coverage over the corresponding region of the phylogenetic tree. Vertical bars indicate broad specificity probes. Striped regions of vertical bars indicate partial coverage over the corresponding region of the phylogenetic tree. Empty diamonds indicate minor lineages opened; their names are shown above the diamonds. Black diamonds indicate major lineages; the names of them are displayed to the right side of the tree (‘Lineage’). On the AOA tree, clusters from a very recent review on AOA phylogeny are also shown in blue [38]. Ns – *Nitrososphaera* subcluster. Np – *Nitrosopumilus* subcluster.
(PDF)

Supporting Information S2 **Microarray probe set.**
(DOCX)

Supporting Information S3 **Representative ARB database.**
(ARB)

Supporting Information S4 **Applicability of alternative** ***amoA*** **PCR primer sets for use with the array.**
(DOC)

Supporting Information S5 **Excel spreadsheets showing weighted mismatch (wMM) values of the probe set against the representative ARB database.** Probes are shown in columns, sequences in rows. For details of wMM calculation, please refer to Experimental Procedures.
(ZIP)

Supporting Information S6 **Excel spreadsheets showing weighted mismatch (wMM) values of the probe set against the full ARB database.** Probes are shown in columns, sequences in rows. For details of wMM calculation, please refer to Experimental Procedures.
(XLSX)

Supporting Information S7 **Evaluation with environmental samples, full results.** Nitrifier community analyses. Results of individual microarray experiments were first normalized to positive control probes, and then to the reference values determined individually for each probe (see Experimental procedures for details), averaged between replicate spots and displayed using the GeneSpring software. In essence, a value of 100% indicates maximum achievable signal for an individual probe, whereas a value of 10% indicates that about 10% of the total PCR product hybridised to that probe. Heat map colour coding is indicated on the side bar. Probes are indicated on the left side of the heat maps, by names and by numbers corresponding to Supporting Information S2. Major clusters are indicated by names and colours (see also Fig. 1; colours correspond between these two figures). A) AOA results; E1–E6: Temperate estuarine sediment samples (Derwent River, Tasmania). E1: upstream; E6: mouth. Os-Ccl: Open ocean water samples (Kimberley region, Western Australia). Os – Open ocean, surface; Ocl – Open ocean, chlorophyll maximum layer; Cs – Coastal area, surface; Ccl – Coastal area, chlorophyll maximum layer. B) AOB results; Sc, St, StN: Agricultural soil samples (Harden, New South Wales). Sc: control; St: tillage treated; StN: tillage treated with nitrogen amendment. W1–W3: Wastewater treatment plant water samples (Sydney, New South Wales). E1–E6: Temperate estuarine sediment samples (Derwent River, Tasmania). E1: upstream; E6: mouth. C) Side bar indicating heat map colour coding. Probe AamoA-159 has been discarded based on evaluation with estuarine sediment samples (see Results for details). The probe is therefore not listed in the final probe set (Supporting Information S1, Supporting Information S2, Supporting Information S5, Supporting Information S6), however is shown here to illustrate the process.
(PDF)

Supporting Information S8 ***amoA*** **array layouts and hybridisation examples.** A. Schematic diagram of the microarray and slide design. Each slide contained three arrays (for three separate assays). Each array consisted of three replicate subarrays. Frames indicate universal probes spotted in multiple copies and spots with an external positive control probe (‘hyaBp’; results of this were not considered or used in the present study). B. Detailed design of a single array with exact positions for each probe. C. Representative hybridisation. Microarray image was adjusted for best viewing (quantitative conclusions drawn from the image may be misleading).
(PDF)
