# Supplementary figures and images for: High-Throughput Analysis of Ammonia Oxidiser Community Composition via a Novel, amoA-Based Functional Gene Array
Source: PLoS One. 2012 Dec 19;7(12):e51542. doi: 10.1371/journal.pone.0051542 (PMC3526613; doi:10.1371/journal.pone.0051542)

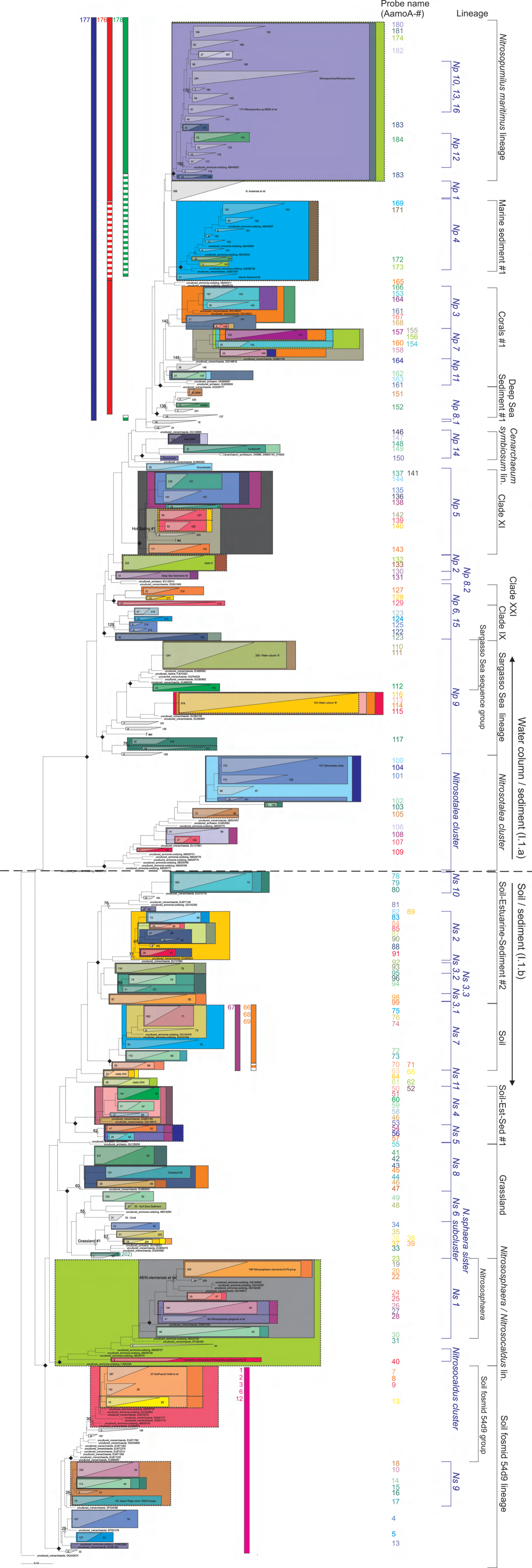

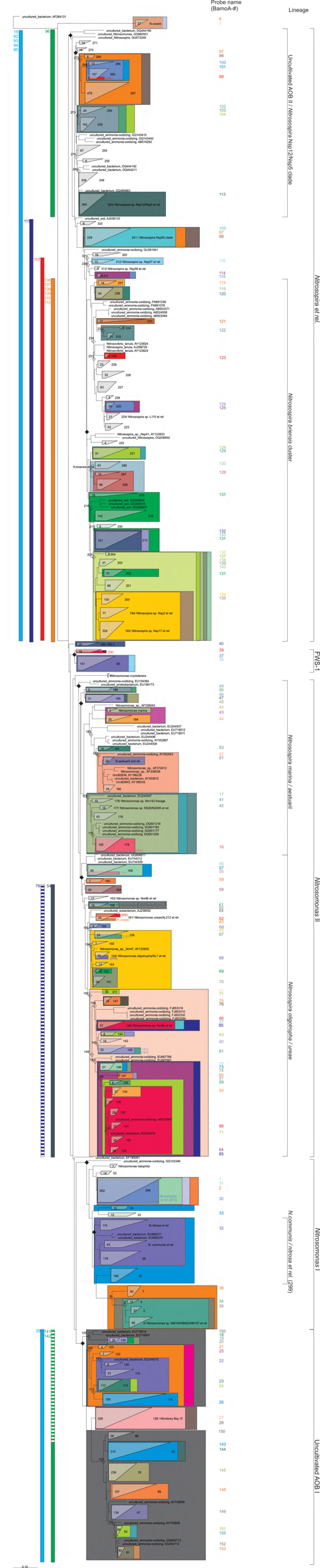

Supplement: Supporting Information S1 — Phylogentic tree with probe specificities (multiple probe concept). Coloured boxes indicate the specificity of the probe on the phylogenetic tree. Corresponding probe names are displayed to the right in the same colour (‘Probe name’). Boxes with dotted lines indicate probes with partial coverage over the corresponding region of the phylogenetic tree. Vertical bars indicate broad specificity probes. Striped regions of vertical bars indicate partial coverage over the corresponding region of the phylogenetic tree. Empty diamonds indicate minor lineages opened; their names are shown above the diamonds. Black diamonds indicate major lineages; the names of them are displayed to the right side of the tree (‘Lineage’). On the AOA tree, clusters from a very recent review on AOA phylogeny are also shown in blue [38]. Ns – Nitrososphaera subcluster. Np – Nitrosopumilus subcluster. (PDF) [file pone.0051542.s001.pdf]
