# Supplementary material for: High-Throughput Analysis of Ammonia Oxidiser Community Composition via a Novel, amoA-Based Functional Gene Array
Source: PLoS One. 2012 Dec 19;7(12):e51542. doi: 10.1371/journal.pone.0051542 (PMC3526613; doi:10.1371/journal.pone.0051542)
Supplement: Supporting Information S2 — Microarray probe set. (DOCX) [file pone.0051542.s002.docx]

##### Supplementary Information 2 – Microarray probe set

| No. | Name | Sequence (5' - 3') | L | GC% | T_m_ |
| --- | --- | --- | --- | --- | --- |
|  | AOA111F | TTYTAYACHGAYTGGGCHTGGACATC | 26 | 46.8 | 58.9 |
|  | AOA643R-c | TGGATGGCCGCYTGGTCSAAGTGGGA | 26 | 63.5 | 68.6 |
|  | Arch-amoAF | STAATGGTCTGGCTTAGACG | 20 | 50.0 | 52.6 |
|  | Arch-amoAR-c | ACATACAGATGGATGGCCGC | 20 | 55.0 | 57.5 |
|  | amoA-23F | ATGGTCTGGCTWAGACG | 17 | 52.9 | 51.2 |
|  | amoA-616R-c | TGGACATACAGATGGATGGC | 20 | 50.0 | 54.4 |
|  |  |  |  |  |  |
| 1 | AamoA-182 | TTGGGATGTACAATGGCAGCATTAGG | 26 | 59.3 | 46.2 |
| 2 | AamoA-183 | CTTCATTGTCACTCCCATTTGGCTACC | 27 | 59.6 | 48.1 |
| 3 | AamoA-184 | TAGCACTCGGAGCCGGTGCAAG | 22 | 63.8 | 63.6 |
| 4 | AamoA-174 | CAAAAGCCGCATGGTTTTCGCTAGGAT | 27 | 61.7 | 48.1 |
| 5 | AamoA-180 | TCTATAACAGCCCAGTTGCTCTCGGTG | 27 | 61.7 | 51.9 |
| 6 | AamoA-181 | AAAGCCGCATGGTTTTCGCTAGGA | 24 | 61.4 | 50.0 |
| 7 | AamoA-172 | CTGATTCTGATGGGCGGAGTATTGGT | 26 | 60.3 | 50.0 |
| 8 | AamoA-173 | GGAGCAGGTGCAGTATTGGGAACATT | 26 | 61.1 | 50.0 |
| 9 | AamoA-169 | TGCTGGTTCGGTATTGGGAGTAACGT | 26 | 61.7 | 50.0 |
| 10 | AamoA-171 | TACTGGTAGGAATGTCGTTACCGATGT | 27 | 58.9 | 44.4 |
| 11 | AamoA-178 | CGTTCCAGGCACAGCAACGTACTA | 24 | 61.1 | 54.2 |
| 12 | AamoA-153 | CTCGCTGATTTTGTTCGGTGGTGTCT | 26 | 61.1 | 50.0 |
| 13 | AamoA-164 | CGGTAGCGCTGGGTGCGGGA | 20 | 67.3 | 75.0 |
| 14 | AamoA-165 | GGTTAACTTGATTACGGTCGCAGACCCA | 28 | 61.9 | 50.0 |
| 15 | AamoA-166 | AACATGGTTAACTTGATTACGGTCGCAG | 28 | 59.1 | 42.9 |
| 16 | AamoA-167 | GCATGTACATTTGCAGCCCTTGGT | 24 | 60.5 | 50.0 |
| 17 | AamoA-168 | CAACATGGTGAATCTCCTAACAGTAGCA | 28 | 58.4 | 42.9 |
| 18 | AamoA-157 | ACGTTTACGGCACTGGGTGCTAAA | 24 | 61.0 | 50.0 |
| 19 | AamoA-156 | CAGGAACAGCTTCGTATTATGGCCTAA | 27 | 58.5 | 44.4 |
| 20 | AamoA-154 | ACATGGTAAACTTGTTGACAGTCGCAG | 27 | 59.3 | 44.4 |
| 21 | AamoA-158 | AACGCCAGTTTGGGCTCCATCATG | 24 | 62.1 | 54.2 |
| 22 | AamoA-160 | AACATGATCAACTTGTTGACGGTCGCAG | 28 | 61.1 | 46.4 |
| 23 | AamoA-155 | ACATGGTACCAATTGAACCGCAGGTC | 26 | 61.2 | 50.0 |
| 24 | AamoA-161 | GCAGTATTGTCGGTAACGTTGGCAGC | 26 | 62.0 | 53.8 |
| 25 | AamoA-162 | TATCGCAAACCTTGATGCTCGTAGTAGG | 28 | 59.8 | 46.4 |
| 26 | AamoA-163 | CTTGATGCTCGTAGTAGGTGCAACCTAT | 28 | 59.7 | 46.4 |
| 27 | AamoA-151 | AAACTTAACACTTGGACGTACCGATGG | 27 | 58.8 | 44.4 |
| 28 | AamoA-152 | TGTCAGTCACTATGGCAGCATTGG | 24 | 59.3 | 50.0 |
| 29 | AamoA-176 | CTTGATACTGTTTGGCGGCGTACT | 24 | 59.3 | 50.0 |
| 30 | AamoA-177 | AACCTGATAACAGTAGCAGACCCACT | 26 | 59.3 | 46.2 |
| 31 | AamoA-146 | ATCGTAGTGCCAGTATGGTTGCCA | 24 | 60.4 | 50.0 |
| 32 | AamoA-147 | GTGCTGTCTTATCAGTAACCTTGGCGG | 27 | 61.0 | 51.9 |
| 33 | AamoA-148 | GTCTGGTTGCCGTCAGGAATGTTACT | 26 | 60.7 | 50.0 |
| 34 | AamoA-149 | CACAGTCTGATACTGTTCGGAGGTGTG | 27 | 60.3 | 51.9 |
| 35 | AamoA-150 | GTGCTGTATTGTCTGTTACGTTGGCGG | 27 | 61.6 | 51.9 |
| 36 | AamoA-144 | TAATTACTGTGGCAGACCCACTGGAA | 26 | 59.7 | 46.2 |
| 37 | AamoA-145 | ATTCATTGATCCTCTTTGGCGGAGTAC | 27 | 58.7 | 44.4 |
| 38 | AamoA-135 | GTCAACCTCATCACTGTGGCTGATCC | 26 | 61.1 | 53.8 |
| 39 | AamoA-136 | CCTCATCACTGTGGCTGATCCATTAGAG | 28 | 60.2 | 50.0 |
| 40 | AamoA-137 | AGACCAACGCTTCCGCCGTATATG | 24 | 61.0 | 54.2 |
| 41 | AamoA-138 | CCGCATTGGGATGCAAACTGAACAC | 25 | 61.1 | 52.0 |
| 42 | AamoA-139 | ATTCGGTGGTGTCATGGCAGGACT | 24 | 62.6 | 54.2 |
| 43 | AamoA-140 | ATTCGGCGGTGT**A**ATGGCAGGACT | 24 | 62.8 | 54.2 |
| 44 | AamoA-143 | TTATGACCGTGTATACATGGGTCGCAAA | 28 | 59.8 | 42.9 |
| 45 | AamoA-142 | GTTGGTAGTTGGCGCAGCTTACTATCT | 27 | 60.4 | 48.1 |
| 46 | AamoA-141 | TCTATTCGGTGGTGTGTTGTGTGGA | 25 | 60.0 | 48.0 |
| 47 | AamoA-132 | ATTACAGTAGCGGATCCATTGGAGAC | 26 | 58.2 | 46.2 |
| 48 | AamoA-133 | GCTGGAGCTGTGTTGTCTGTAACGT | 25 | 61.1 | 52.0 |
| 49 | AamoA-130 | CAGGGACAGCTACTTACTACGCGTTA | 26 | 59.6 | 50.0 |
| 50 | AamoA-131 | ACTGTGTATACTTGGGTAGCAAAGGGCG | 28 | 61.9 | 50.0 |
| 51 | AamoA-127 | ACATTTACTGGTGTTCCAGGGACC | 24 | 58.9 | 50.0 |
| 52 | AamoA-128 | TCTTGTCGGTTACGTTCGCGGCAT | 24 | 62.9 | 54.2 |
| 53 | AamoA-129 | TTGATGCTAGCTGTTGGAGCTTCGTA | 26 | 60.0 | 46.2 |
| 54 | AamoA-123 | CAGGTGCTGGAGCTGTTTTGTCGGTTA | 27 | 62.7 | 51.9 |
| 55 | AamoA-124 | GGTGGAGTTATGGTCGGAATGTCG | 24 | 59.4 | 54.2 |
| 56 | AamoA-125 | GTAATGGTCGGAATGTCGTTGCCGTT | 26 | 61.3 | 50.0 |
| 57 | AamoA-121 | AACGTTTACAGGCGTTGTGGGAAC | 24 | 60.1 | 50.0 |
| 58 | AamoA-122 | TTACAGGCGTTGTGGGAACAGCGA | 24 | 62.9 | 54.2 |
| 59 | AamoA-110 | CAATTTGATCACAGTGGCTGACCC | 24 | 58.5 | 50.0 |
| 60 | AamoA-111 | CGGCGGAGTCTTAGTTGGAATGTC | 24 | 59.5 | 54.2 |
| 61 | AamoA-112 | CAGGTACGGCGACATATTATGCGC | 24 | 60.0 | 54.2 |
| 62 | AamoA-113 | AGTAGCTTTGGGAGCGGGAGCT | 22 | 62.8 | 59.1 |
| 63 | AamoA-116 | CTTTGGAGGAGTTTTGGTCGGTATGTC | 27 | 59.2 | 48.1 |
| 64 | AamoA-114 | TTCATCGTTCATCCGATGTGGATTCC | 26 | 58.9 | 46.2 |
| 65 | AamoA-115 | TGATTGCGGATCCGCTAGAAACTG | 24 | 59.2 | 50.0 |
| 66 | AamoA-120 | GAGCTGGTGCTGTCCTGGCA | 20 | 62.6 | 65.0 |
| 67 | AamoA-119 | ACTTTATCGTGACCCCGGTTTGGT | 24 | 60.6 | 50.0 |
| 68 | AamoA-118 | CGGAGTATTGGTAGGTATGTCGCTACC | 27 | 59.7 | 51.9 |
| 69 | AamoA-117 | AGTATTAGCCGTTACGATGGCAGC | 24 | 59.0 | 50.0 |
| 70 | AamoA-101 | AAGGTGCGTGGTTCTCCTTAGGATATC | 27 | 59.8 | 48.1 |
| 71 | AamoA-100 | AATCATTGATGCTCGCAGTCGGTG | 24 | 59.8 | 50.0 |
| 72 | AamoA-104 | CACAATCATTGATGCTCGCAGTCG | 24 | 58.7 | 50.0 |
| 73 | AamoA-102 | ACAATGGCTGCCTTGGGCACAAAG | 24 | 62.8 | 54.2 |
| 74 | AamoA-103 | CTCCTTGGGGTACCCGTACAGTTTC | 25 | 60.7 | 56.0 |
| 75 | AamoA-105 | CATGCTTGCTGTCGGTGCCGCGTA | 24 | 62.5 | 66.0 |
| 76 | AamoA-107 | CCTAATGGGTGGTGTGCTCGTAGG | 24 | 61.2 | 58.3 |
| 77 | AamoA-106 | AATCTCTTGCTGGTTGCTGATCCG | 24 | 59.5 | 50.0 |
| 78 | AamoA-108 | TCAGTGACGATGACAGCGTTAGGC | 24 | 60.9 | 54.2 |
| 79 | AamoA-109 | TCACAATCACTCATGCTAGCAGTCG | 25 | 58.8 | 48.0 |
| 80 | AamoA-78 | AGCGGTGCTGAGCGTGCCTAT | 21 | 63.9 | 61.9 |
| 81 | AamoA-79 | AACGCTCGTTGGACTGTCGCTG | 22 | 62.1 | 59.1 |
| 82 | AamoA-80 | TTACTGGAGTCCCTGGAACGGC | 22 | 61.0 | 59.1 |
| 83 | AamoA-81 | AACACTGGTTGGGCTGTCACTACC | 24 | 61.1 | 54.2 |
| 84 | AamoA-82 | AGTGCTAAGTGTGCCTATTGCGGC | 24 | 61.7 | 54.2 |
| 85 | AamoA-83 | TTGGCGGAACACTGGTAGGTTTGT | 24 | 60.9 | 50.0 |
| 86 | AamoA-84 | GGTTGGCTTGTCGCTGCCCAT | 21 | 63.3 | 61.9 |
| 87 | AamoA-85 | CAGTTCTGACTGTGCCAATTGCGG | 24 | 60.8 | 54.2 |
| 88 | AamoA-88 | ATGACAATATACACCTGGGTGGCC | 24 | 58.9 | 50.0 |
| 89 | AamoA-87 | AACTTTGGTGGGTTTGTCGCTGCC | 24 | 62.6 | 54.2 |
| 90 | AamoA-90 | TCGGCGATGTTGTTAGATCTCACG | 24 | 58.8 | 50.0 |
| 91 | AamoA-91 | GCTGTTCCCGTATGGATACCGTCT | 24 | 60.3 | 54.2 |
| 92 | AamoA-89 | CCGATTGCGGCATTGGGTG | 19 | 60.0 | 63.2 |
| 93 | AamoA-92 | AGGATACCCAATGGACTTCGTAACAG | 26 | 58.1 | 46.2 |
| 94 | AamoA-93 | GCGCAATAAACACGCCGCCATTAT | 24 | 60.9 | 50.0 |
| 95 | AamoA-94 | TCCTCTCGAGGTCGCGTTCAAGTA | 24 | 61.3 | 54.2 |
| 96 | AamoA-95 | TAAGGGATCCTCTCGAGGTCGCGT | 24 | 63.0 | 58.3 |
| 97 | AamoA-96 | TGGTAAGGGATCCTCTCGAGGTCG | 24 | 61.3 | 58.3 |
| 98 | AamoA-98 | CTCGAAGTCGCCTTTAAGTATCCGAG | 26 | 58.6 | 50.0 |
| 99 | AamoA-99 | GTCGCCTTTAAGTATCCGAGACCC | 24 | 58.9 | 54.2 |
| 100 | AamoA-74 | AGCAGGCGCTTCAGCAGTGCTA | 22 | 63.7 | 59.1 |
| 101 | AamoA-76 | CTGCTAGTGCGAGATCCGTTGGAA | 24 | 60.8 | 54.2 |
| 102 | AamoA-75 | GCTACGCGGCGCAATAAGCACA | 22 | 62.9 | 59.1 |
| 103 | AamoA-72 | AATACTCGTTGGCGGCACTTTGGT | 24 | 61.3 | 50.0 |
| 104 | AamoA-73 | TGCTAATACTCGTTGGCGGCACTTTG | 26 | 61.4 | 50.0 |
| 105 | AamoA-70 | AACGCGGCGGAACAAACACAT | 21 | 60.7 | 52.4 |
| 106 | AamoA-71 | GCGGTGCTGACAGTACCTATAGCA | 24 | 60.4 | 54.2 |
| 107 | AamoA-63 | GCGCAATAAGCACGCCGCTATACT | 24 | 61.8 | 54.2 |
| 108 | AamoA-64 | GGAACTTTGATCGGTCTCTCAATCCCAC | 28 | 60.6 | 50.0 |
| 109 | AamoA-65 | TGCTGGTAAGAGATCCCCTGGAAGTAG | 27 | 60.9 | 51.9 |
| 110 | AamoA-66 | GTGCGAGATCCGTTGGAAGTCGCA | 24 | 63.3 | 58.3 |
| 111 | AamoA-68 | GCTACGCGGCGCAATAAGCACATG | 24 | 63.1 | 58.3 |
| 112 | AamoA-69 | TGCTAATACTTGTCGGCGGTACCTTG | 26 | 60.5 | 50.0 |
| 113 | AamoA-67 | CGAGATCCGTTGGAAGTCGCATTC | 24 | 59.9 | 54.2 |
| 114 | AamoA-61 | TGATAAACTTGTTGCTGGTGCGGG | 24 | 60.1 | 50.0 |
| 115 | AamoA-62 | GGGCCACAAGGCGGAACAAACATG | 24 | 63.3 | 58.3 |
| 116 | AamoA-59 | ATTGACCATACGCGACCCGTTAGA | 24 | 60.0 | 50.0 |
| 117 | AamoA-60 | AAGGAACAAACACGCGGCGATCCT | 24 | 63.3 | 54.2 |
| 118 | AamoA-50 | ACGATGCTTGTTGTGGGTGCCATC | 24 | 62.2 | 54.2 |
| 119 | AamoA-58 | GAACGTTAGTTGGACTCTCGCTGC | 24 | 59.7 | 54.2 |
| 120 | AamoA-46 | AACACGCGGCGATAATCATTGGAG | 24 | 59.8 | 50.0 |
| 121 | AamoA-51 | ACATGTTGTTTACGGGAGTGCCAG | 24 | 59.6 | 50.0 |
| 122 | AamoA-52 | GAGTGCCAGGGACAGCGACCTA | 22 | 63.0 | 63.6 |
| 123 | AamoA-57 | ATCCACGTCCGACATTACCGCC | 22 | 61.5 | 59.1 |
| 124 | AamoA-55 | CATAGGCGGGACACTCGTAGGTTT | 24 | 60.5 | 54.2 |
| 125 | AamoA-53 | GAGTGCCGGGTACGGCTACT | 20 | 61.4 | 65.0 |
| 126 | AamoA-54 | GCCGGGTACGGCTACTTATTATGC | 24 | 59.5 | 54.2 |
| 127 | AamoA-56 | CCTGACTTATTGGGCGACGAGAAG | 24 | 59.3 | 54.2 |
| 128 | AamoA-41 | AGCACGCAGCGATAATAATAGGCGG | 25 | 61.1 | 52.0 |
| 129 | AamoA-42 | ACTTAGTGTACCGATTGCTGCATTGGG | 27 | 60.8 | 48.1 |
| 130 | AamoA-44 | CACTACCTCCGTATATGACCCCGA | 24 | 59.3 | 54.2 |
| 131 | AamoA-47 | TACTTAGCGTACCTATTGCAGCATTAGG | 28 | 58.0 | 42.9 |
| 132 | AamoA-43 | CTACACTTGGGTGGCGAAGGGT | 22 | 61.4 | 59.1 |
| 133 | AamoA-45 | GATCTTACTTACTGGGCCACTAGGAGA | 27 | 58.8 | 48.1 |
| 134 | AamoA-49 | AGCATGCAGCCATTCTAATAGGTGGA | 26 | 60.1 | 46.2 |
| 135 | AamoA-48 | AGCTGGGGCAGTGCTTAGTGTTC | 23 | 61.6 | 56.5 |
| 136 | AamoA-34 | TGTTTACCGGTGTCCCTGGCAC | 22 | 61.8 | 59.1 |
| 137 | AamoA-35 | TATTTACGGGTGTTCCTGGAACGGCT | 26 | 61.8 | 50.0 |
| 138 | AamoA-36 | CTTTGTGTTCCTATTGCGGCCTTGG | 25 | 60.5 | 52.0 |
| 139 | AamoA-37 | AGCAATACTAATTGGAGGAGTGCTGGT | 27 | 59.7 | 44.4 |
| 140 | AamoA-38 | GCAATACTAATTGGAGGAGTGCTGGT | 26 | 58.8 | 46.2 |
| 141 | AamoA-39 | CAATACTAATTGGAGGAGTGCTGGT | 25 | 56.6 | 44.0 |
| 142 | AamoA-33 | ACGCGGCAATTATCATTGGAGGCA | 24 | 61.5 | 50.0 |
| 143 | AamoA-20 | GTGCAGTGCTAACGGTGCCCAT | 22 | 62.5 | 59.1 |
| 144 | AamoA-22 | GTGCAGTGCTAACGGTGCCCAT | 22 | 62.5 | 59.1 |
| 145 | AamoA-24 | CCATACTGATTGGTGGAACTATGGTTGG | 28 | 59.0 | 46.4 |
| 146 | AamoA-25 | ACTGACAGTCCCCATGGCAGC | 21 | 62.1 | 61.9 |
| 147 | AamoA-26 | GGCTACAAGGCGGAACAAGCAC | 22 | 61.1 | 59.1 |
| 148 | AamoA-27 | GCACGCTGCAATCCTCATCGGT | 22 | 62.5 | 59.1 |
| 149 | AamoA-28 | ACTGATTGGCCTCTCGTTGCCG | 22 | 62.0 | 59.1 |
| 150 | AamoA-19 | GCTGTTCAACATGGTCAACCTGTTGC | 26 | 60.8 | 50.0 |
| 151 | AamoA-30 | GGTTCCAATAGCTGCGTTAGGAGCAAAG | 28 | 61.3 | 50.0 |
| 152 | AamoA-31 | TCTCTCAATCTACTATGCTCGCAGTGGG | 28 | 60.9 | 50.0 |
| 153 | AamoA-23 | CGGCCACATACTACGCCACAATCATG | 26 | 61.6 | 53.8 |
| 154 | AamoA-40 | ACCTATTGAACCACAGGTGGGGAA | 24 | 60.1 | 50.0 |
| 155 | AamoA-10 | GTTTGCGCTAGGATATCCAATGGACT | 26 | 58.8 | 46.2 |
| 156 | AamoA-18 | TACTAAGTGTTCCAATAGCTGCGCTAGG | 28 | 59.7 | 46.4 |
| 157 | AamoA-14 | ACGATTTCGTCTTAGTACCTGTTTGG | 26 | 56.8 | 42.3 |
| 158 | AamoA-15 | GATTTCGTCTTAGTACCTGTTTGGATAC | 28 | 55.3 | 39.3 |
| 159 | AamoA-16 | CGATTTCGTCTTAGTACCTGTTTGGATA | 28 | 56.3 | 39.3 |
| 160 | AamoA-17 | GCTGGTCAGAGATCCGTTAGAAATGG | 26 | 59.0 | 50.0 |
| 161 | AamoA-13 | CGACTTTATCGCCGTACCAGTCTGGAT | 27 | 61.5 | 51.9 |
| 162 | AamoA-7 | GAGCGTTCCAATCGCTGCATTGGG | 24 | 62.9 | 58.3 |
| 163 | AamoA-8 | CTGAGCGTTCCAATCGCTGCATTG | 24 | 61.0 | 54.2 |
| 164 | AamoA-9 | CGACTTCGTGGTAACTCCAGTCTG | 24 | 58.9 | 54.2 |
| 165 | AamoA-4 | GGAACGCTGGTTGGTCTTTCATTG | 24 | 62.5 | 53.8 |
| 166 | AamoA-5 | TGATCAATCTGATTCTGGTAAGAGATCC | 28 | 56.0 | 39.3 |
| 167 | AamoA-1 | TCACCGGTGTACCAGGGACT | 20 | 60.2 | 60.0 |
| 168 | AamoA-2 | ATAGTCCCGTGGCGCTAGGATC | 22 | 60.6 | 59.1 |
| 169 | AamoA-3 | GCTTGTAGTTGGTGCAATCTATTACATGC | 29 | 58.2 | 41.4 |
| 170 | AamoA-6 | GAAGGAATAAACACGCTGCCATTATTAT | 28 | 55.9 | 35.7 |
| 171 | AamoA-12 | AGTCGGTGCAATCTATTACATGCTGT | 26 | 58.3 | 42.3 |
|  |  |  |  |  |  |
|  | amoA-1F | GGGGTTTCTACTGGTGGT | 18 | 55.6 | 54.1 |
|  | amoAf-i | GGGGITTITACTGGTGGT | 18 | 50.0 | 56.3 |
|  | amoA-2R-c | GAAGAAGGCTTTSCMGAGGGG | 21 | 59.5 | 59.2 |
|  | amoAr-i-c | GAAGAAGGITTTICIGAGGGG | 21 | 47.6 | 59.7 |
|  | mtrof173a | GGGGACTGGGACTTCTGG | 18 | 66.7 | 57.5 |
|  | mtrof662-I | GGTAAGGACGTTGCGCCGG | 19 | 68.4 | 61.6 |
|  | pmoA682-C | GCSTTCTTCTCNGCSTTC | 18 | 58.3 | 54.9 |
|  | AMO-F | AGAAATCCTGAAAGCGGC | 18 | 50.0 | 53.0 |
|  | AMO-R-c | CTTCTCTGCGTTCGTATC | 18 | 50.0 | 50.1 |
|  |  |  |  |  |  |
| 1 | BamoA-6 | TTCGTAGGGTTTACCTATTTTCCGATTA | 28 | 55.8 | 35.7 |
| 2 | BamoA-7 | GGATGCCATGCTTGCGATTTCCAA | 24 | 60.7 | 50.0 |
| 3 | BamoA-99 | CTCTATGTACGCACAGGCACGCC | 23 | 62.0 | 60.9 |
| 4 | BamoA-100 | TCATTGCGCACCTTCGGGGGACAC | 24 | 65.7 | 62.5 |
| 5 | BamoA-101 | CATTGCGCACCTTCGGGGGAC | 21 | 63.6 | 66.7 |
| 6 | BamoA-97 | GGCGTATTGCTCTCCCTGGC | 20 | 60.8 | 65.0 |
| 7 | BamoA-98 | CGAAGGTGTATTGCTCTCCCTGG | 23 | 59.4 | 56.5 |
| 8 | BamoA-102 | GAAGGCGTATTACTGTCCCCGGCT | 24 | 62.7 | 58.3 |
| 9 | BamoA-103 | GCGTATTACTGTCCCCGGCTG | 21 | 61.9 | 60.2 |
| 10 | BamoA-104 | TGCCGCTGGTAGCAGAAGGCGTATTA | 26 | 63.7 | 53.8 |
| 11 | BamoA-113 | CCGAAGGCGTCCTCTTGTCCCT | 22 | 63.3 | 63.6 |
| 12 | BamoA-105 | GGCTGAAGGCGTCCTGCTCTCC | 22 | 64.4 | 68.2 |
| 13 | BamoA-96 | CGGAATACGTCCGGCTGATCGA | 22 | 60.9 | 59.1 |
| 14 | BamoA-114 | GCGGGCGCATGACCATGAA | 19 | 61.5 | 63.2 |
| 15 | BamoA-115 | GGCGCATGACCATGAAAAACGACG | 24 | 61.3 | 54.2 |
| 16 | BamoA-116 | CTCACTGGCCGACTACACAGGC | 22 | 61.9 | 63.3 |
| 17 | BamoA-118 | CGGGTACCCCTGAATATGTGCGC | 23 | 62.1 | 60.9 |
| 18 | BamoA-119 | CGTTCTCCTGTCGGTGGCTGAC | 22 | 62.0 | 63.6 |
| 19 | BamoA-120 | CGAAGGCGTTCTCCTGTCGGTG | 22 | 62.2 | 63.6 |
| 20 | BamoA-121 | TGATCGGCGGGGGTGCCTTT | 20 | 64.6 | 65.0 |
| 21 | BamoA-122 | ATCGCTACGAACGTTCGGAGGC | 22 | 61.5 | 59.1 |
| 22 | BamoA-123 | ACACCGTCCTCTTGCTCACGC | 21 | 62.1 | 61.9 |
| 23 | BamoA-124 | GCGGCGCATTCGGGTTACTGT | 21 | 62.9 | 61.9 |
| 24 | BamoA-125 | CGTATTACTGTCGGTTGCCGACTAC | 25 | 59.0 | 52.0 |
| 25 | BamoA-128 | CCTGAGTATGTCCGCAACATCGAACA | 26 | 60.6 | 50.0 |
| 26 | BamoA-129 | ACGCCTGAGTATGTCCGCAACA | 22 | 61.1 | 54.5 |
| 27 | BamoA-126 | CCCCGAGTACGTACGCAACATCGA | 24 | 62.4 | 58.3 |
| 28 | BamoA-130 | CTGCTCTCCGTTGCCGACTACA | 22 | 61.1 | 59.1 |
| 29 | BamoA-131 | AGCATGAAGAACGACGTGACAGCA | 24 | 60.9 | 50.0 |
| 30 | BamoA-132 | ACTGGACCATATTTGGCCCCACCC | 24 | 63.4 | 58.3 |
| 31 | BamoA-133 | GCTGGTGGCAGAAGGCGTATTGCT | 24 | 61.5 | 54.5 |
| 32 | BamoA-134 | TGGTTACAGCCCTGATTGGCGGGG | 24 | 65.5 | 62.5 |
| 33 | BamoA-135 | GGATGGTTACAGCCCTGATTGGCG | 24 | 62.1 | 58.3 |
| 34 | BamoA-136 | TCTACTACGTAAAAGGCGCCCGTG | 24 | 60.7 | 54.2 |
| 35 | BamoA-137 | CAACTGGATGGTTACAGCCCTG | 22 | 58.0 | 54.5 |
| 36 | BamoA-138 | GCAACTGGATGGTTACAGCCCTGAT | 25 | 61.1 | 52.0 |
| 37 | BamoA-139 | CTTCTACTACGTAAAAGGCGCCCG | 24 | 59.4 | 54.2 |
| 38 | BamoA-140 | AACTGGATGGTTACAGCCCTGATTGG | 26 | 60.8 | 50.0 |
| 39 | BamoA-106 | ACGCCTGAGTACGTGCGCCTGA | 22 | 65.2 | 63.6 |
| 40 | BamoA-107 | ACGCCTGAGTACGTGCGCCT | 20 | 64.0 | 65.0 |
| 41 | BamoA-108 | CAACTGGATGGTTACAGCCCTGAT | 24 | 59.0 | 50.0 |
| 42 | BamoA-109 | CAACTGGATGGTTACAGCCCTGATTG | 26 | 59.7 | 50.0 |
| 43 | BamoA-110 | TCTCCATGCTCATGTTCACCGT | 22 | 58.6 | 50.0 |
| 44 | BamoA-112 | GCTGAAGGCGTACTGCTGTCGGTAGC | 26 | 64.8 | 61.5 |
| 45 | BamoA-117 | TCGGTAGCCGACTACACCGGCT | 22 | 64.3 | 63.6 |
| 46 | BamoA-111 | GGCTGAAGGCGTCCTGCTCTCC | 22 | 64.4 | 68.2 |
| 47 | BamoA-15 | GCTGGTCATGGACACCGTCTTG | 22 | 60.4 | 59.1 |
| 48 | BamoA-92 | TCGTCTCCATGCTCATGTTCTGCG | 24 | 61.2 | 54.2 |
| 49 | BamoA-93 | GTCATGCTGCTCACCCGCAACT | 22 | 62.5 | 22.0 |
| 50 | BamoA-94 | TGCCGCTGGTAGCCGAAGG | 19 | 62.9 | 68.4 |
| 51 | BamoA-95 | GAGTACGTGCGCCTGATCGAAC | 22 | 60.1 | 59.1 |
| 52 | BamoA-40 | AGGCCGTGTATCGATGAAAGAAGA | 24 | 58.0 | 45.8 |
| 53 | BamoA-39 | CGCTTTGCGTAGTTTGCTTATTGGTT | 26 | 58.6 | 42.3 |
| 54 | BamoA-37 | GAAGGGGTCTTGCTGTCGGTTG | 22 | 60.4 | 59.1 |
| 55 | BamoA-38 | ACCTGTCTGTTGCTGACCCGCA | 22 | 63.4 | 59.1 |
| 56 | BamoA-49 | AGAGAGGTCGTATATCAATAAGGCATGA | 28 | 56.9 | 39.3 |
| 57 | BamoA-50 | TCGTAGTTGAAGGTGTTCTTCTTTCAGT | 28 | 57.8 | 39.3 |
| 58 | BamoA-46 | ACTATGTTAAAGGCGAGCGTGGC | 23 | 59.7 | 52.2 |
| 59 | BamoA-47 | CACTCGTTGTTGAAGGCGTTTTACT | 25 | 57.9 | 44.0 |
| 60 | BamoA-48 | GCATTTCAATGAAGAAAGACGTTACAGC | 28 | 57.0 | 39.3 |
| 61 | BamoA-45 | AGTTGTAGAAGGGGTACTGTTGTCAG | 26 | 58.0 | 46.2 |
| 62 | BamoA-44 | CATCAACGATGATTCCAGGTGCACTG | 26 | 59.9 | 50.0 |
| 63 | BamoA-43 | GGTACACCGGAGTATGTGAGGCTG | 24 | 60.9 | 58.3 |
| 64 | BamoA-53 | AACCCATTTGCCGGTGGTTGTAG | 23 | 60.1 | 52.2 |
| 65 | BamoA-51 | GATTACAGCGTTACTGGGTGGTGG | 24 | 59.6 | 54.2 |
| 66 | BamoA-52 | TTGATTGAACAGGGATCGTTACGAACG | 27 | 58.9 | 44.4 |
| 67 | BamoA-17 | CTTTCAGTTGCTGACTACACTGGTTT | 26 | 57.4 | 42.3 |
| 68 | BamoA-41 | CTGGGGTCTGTTCTTTTACCCTGG | 24 | 59.3 | 54.2 |
| 69 | BamoA-42 | TGGTGGTTTCTGGGGTCTGTTCTTTTAC | 28 | 60.5 | 46.4 |
| 70 | BamoA-16 | CCTTGGTAAGGTTTACTGCACGGC | 24 | 60.2 | 54.2 |
| 71 | BamoA-55 | ACGGGTACACCGGAATACGTAAGATT | 26 | 59.4 | 46.2 |
| 72 | BamoA-56 | ACCATATTGCTGCTGACCGGTAAC | 24 | 59.3 | 50.0 |
| 73 | BamoA-57 | ACCATATTGCTGCTGACCGGTAACT | 25 | 60.3 | 48.0 |
| 74 | BamoA-59 | CTGGACACCATTATGCTGTTAACGGG | 26 | 59.6 | 50.0 |
| 75 | BamoA-58 | CCGGCAACTGGTTAGTGACAGC | 22 | 60.6 | 59.1 |
| 76 | BamoA-61 | GTACTGGTACGCCAGAATATGTTCGC | 26 | 59.2 | 50.0 |
| 77 | BamoA-60 | TACCCGTGGTCGTTGAAGGTGT | 22 | 60.5 | 54.5 |
| 78 | BamoA-64 | GTTATACTGTACCGCTTTCTTCTATGTT | 28 | 54.8 | 35.7 |
| 79 | BamoA-62 | CTTTGGCAAGTTATACTGTACCGCTTTC | 28 | 57.9 | 42.9 |
| 80 | BamoA-63 | ATTGCTGCGTTTTTCTCAGCCTTTG | 25 | 58.9 | 44.0 |
| 81 | BamoA-66 | TCAATCGCTGACTACACAGGCT | 22 | 58.3 | 50.0 |
| 82 | BamoA-67 | ACAGCGCTGTTAGGAGGAGGATTT | 24 | 60.2 | 50.0 |
| 83 | BamoA-68 | CAGTGGTAGTTGAAGGGGTATTACTGTC | 28 | 58.3 | 46.4 |
| 84 | BamoA-69 | CCAGGTGCATTGATGATGGATACAATCA | 28 | 59.0 | 42.9 |
| 85 | BamoA-70 | GATTCCAGGTGCATTGATGATGGACA | 26 | 59.2 | 46.2 |
| 86 | BamoA-65 | GAAAGAGGACGCATATCAATGAAGAACG | 28 | 57.7 | 42.9 |
| 87 | BamoA-75 | GCTGATGCTGGACACGATTTTGTTATTG | 28 | 58.7 | 42.9 |
| 88 | BamoA-76 | TGGTAACGGCACTACTGGGTGG | 22 | 61.2 | 59.1 |
| 89 | BamoA-77 | GGCCTTCTTTGCAGCTTTCGTATC | 24 | 58.6 | 50.0 |
| 90 | BamoA-83 | GGTTCTTGTACGTCCGTACGGGT | 23 | 60.6 | 56.5 |
| 91 | BamoA-80 | AGATACGATTATGTTGTTGACAGGTAAC | 28 | 55.0 | 35.7 |
| 92 | BamoA-81 | TTCTATGAAGAATGACGTAACTGCTTTT | 28 | 55.0 | 32.1 |
| 93 | BamoA-72 | TTATGTATTGTTCAGCCCTGATGTATTA | 28 | 54.3 | 32.1 |
| 94 | BamoA-73 | TAACACCGATTGTAGGCATTATGTATTG | 28 | 55.3 | 35.7 |
| 95 | BamoA-74 | CGCTGACTACACCGGATTTCTGTACGTA | 28 | 61.2 | 50.0 |
| 96 | BamoA-90 | GCGCTGATGTTAGATACGATTTTATTGT | 28 | 55.8 | 35.7 |
| 97 | BamoA-91 | GATTGATTGAACAAGGATCACTGCGAAC | 28 | 58.3 | 42.9 |
| 98 | BamoA-89 | CACGGTTATTGCAGCATTCTTTGGAGCC | 28 | 62.0 | 50.0 |
| 99 | BamoA-88 | CACAGGATTTCTGTATGTGCGGACAG | 26 | 59.5 | 50.0 |
| 100 | BamoA-71 | TGTTAGATACAATTTTACTGTTGACGGG | 28 | 55.3 | 35.7 |
| 101 | BamoA-86 | GTGATTGCGGCCTTCTTTGGAGC | 23 | 61.1 | 56.5 |
| 102 | BamoA-85 | CTTACCAGTGGTAGTAGAAGGTGTATTA | 28 | 55.3 | 39.3 |
| 103 | BamoA-84 | TTCTGGGGATTGTTTTTCTATCCAGG | 26 | 56.9 | 42.3 |
| 104 | BamoA-82 | ATGAAGTACGACGTAACAGCTTTTGGT | 27 | 58.5 | 40.7 |
| 105 | BamoA-54 | ACCCACTTACCAGTGGTAGTAGAAGG | 26 | 59.2 | 50.0 |
| 106 | BamoA-79 | TTTGGTCCAACCCACTTACCAGTG | 24 | 59.3 | 50.0 |
| 107 | BamoA-30 | ACCAGAGTATGTTCGTCATATTGAGCA | 27 | 57.7 | 40.7 |
| 108 | BamoA-31 | GGTGGATTCTTCGGTCTGCTGTTCT | 25 | 60.7 | 52.0 |
| 109 | BamoA-1 | CTGACTACATGGGCCATATGTATGTTCG | 28 | 58.7 | 48.0 |
| 110 | BamoA-2 | AAGGAACACTGTTGTCGATGGC | 22 | 57.8 | 50.0 |
| 111 | BamoA-32 | CAATGGCAGACTACATGGGACACC | 24 | 59.7 | 54.2 |
| 112 | BamoA-33 | GTGCAAAGAGATGACGTTACCGC | 23 | 58.5 | 52.2 |
| 113 | BamoA-34 | TTGATTGAGAAAGGGTCATTACGTACCT | 28 | 57.7 | 39.3 |
| 114 | BamoA-35 | GCCGATTGTAGTAGAAGGGCACTTATT | 27 | 58.5 | 44.4 |
| 115 | BamoA-36 | GTACATAGAGAAGACGTGACTGCATTT | 27 | 56.6 | 40.7 |
| 116 | BamoA-18 | GATTAACTTTGTAGTGCCTTCCACAATG | 28 | 56.4 | 39.3 |
| 117 | BamoA-19 | GTCCAAGAGGCAAGATAACAGAGAAGA | 27 | 58.0 | 44.4 |
| 118 | BamoA-20 | TTGGAAGATTCTACTGTACAGCGTTCT | 27 | 57.6 | 40.7 |
| 119 | BamoA-26 | CGGCGGATGTTTTGGACTGTTC | 22 | 58.9 | 54.5 |
| 120 | BamoA-23 | TACTTAGGAGCGTTCTATTGCACCG | 25 | 58.4 | 48.0 |
| 121 | BamoA-24 | ACAGCGTTGCTTGGTGGTGGAT | 22 | 61.9 | 54.5 |
| 122 | BamoA-22 | TCTCTGGTGGTACTTAGGAGCGTT | 24 | 59.2 | 50.0 |
| 123 | BamoA-21 | ATGATTCCTGGGGCATTGATGATGG | 25 | 59.4 | 48.0 |
| 124 | BamoA-25 | ACTGGATGATTACAGCGTTGCTTGG | 25 | 59.5 | 48.0 |
| 125 | BamoA-27 | GCCATCCACGATGATTCCGGGAG | 23 | 61.9 | 60.9 |
| 126 | BamoA-28 | TGGCGGAGCATTTGGGCTATTG | 22 | 60.4 | 54.5 |
| 127 | BamoA-143 | CATGGATACATGTCTACTGTTGACCCGT | 28 | 59.8 | 46.4 |
| 128 | BamoA-144 | GCGCTGATCATGGATACATGTCTACTGT | 28 | 60.0 | 46.4 |
| 129 | BamoA-145 | GCTGATCATGGACACAGTTCTGTTGT | 26 | 59.2 | 46.2 |
| 130 | BamoA-148 | TCTGGTGGTATCTGGGAAGATTCTATTG | 28 | 57.6 | 42.9 |
| 131 | BamoA-149 | AGAAGGAGTGTTACTTTCGTTAGCTGAC | 28 | 58.2 | 42.9 |
| 132 | BamoA-151 | CATGTCTTTTGCTGACCCGTAACTGG | 26 | 59.9 | 50.0 |
| 133 | BamoA-155 | ACACATGTCTTTTGCTGACCCGTAACT | 27 | 60.4 | 44.4 |
| 134 | BamoA-152 | TTTGGACTACTATTCTATCCGGGTAACT | 28 | 56.7 | 39.3 |
| 135 | BamoA-153 | GACACAACTCTGTTGCTGACCCGTAA | 26 | 60.7 | 50.0 |
| 136 | BamoA-150 | TCGTTGGCTGACTATACCGGATTCCTTT | 28 | 61.1 | 46.4 |
| 137 | BamoA-141 | GACCGACTCACCTCCCGTTAGTA | 23 | 59.6 | 56.5 |
| 138 | BamoA-142 | CACCTCCCGTTAGTAGTAGAAGGAGT | 26 | 58.7 | 50.0 |
| 139 | BamoA-29 | TGGTGGTACCTGGGAAGGTTCT | 22 | 60.0 | 54.5 |
|  |  |  |  |  |  |
|  | hyaBp2 | GATTACGCGCATCGAAGGCCACAT | 24 | 54.2 | 61.9 |
