# Supplementary material for: High-Throughput Analysis of Ammonia Oxidiser Community Composition via a Novel, amoA-Based Functional Gene Array
Source: PLoS One. 2012 Dec 19;7(12):e51542. doi: 10.1371/journal.pone.0051542 (PMC3526613; doi:10.1371/journal.pone.0051542)
Supplement: Supporting Information S4 — Applicability of alternative amoA PCR primer sets for use with the array. (DOC) [file pone.0051542.s004.doc]

| **Applicability** | **Primer** | **Sequence** | **Reference** | **Position in ARB database** | **Direction** |
| --- | --- | --- | --- | --- | --- |
| ***Bacteria*** |  |  |  |  |  |
| Y | amoA21f(AMO-F2) | AGA AAT CCT GAA AGC GGC |  | 973 | Fw |
| Y | amoA34f | GCG GCR AAA ATG CCG CCG GAA GCG |  | 1003 | Fw |
| Y | amoA40f (AMO-F2) | AAG ATG CCG CCG GAA GC |  | 1009 | Fw |
| Y | amoA49f | GAG GAA GCT GCT AAA GTC |  | 1018 | Fw |
| n | amoA60r (304R) | TAY CGC TTC CGG CGG CAT TTT CGC CGC |  | 1003 | Rev |
| Y | amoA121f (amoA-3F) | ACC TAC CAC ATG CAC TT |  | 1090 | Fw |
| Y | amoA151f (A189) | GGN GAC TGG GAC TTCTGG |  | 1120 | Fw |
| Y | amoA154f (301F) | GAC TGG GAC TTC TGG CTG GAC TGG AA |  | 1123 | Fw |
| Y | amoA154fs | GAC TGG GAC TTC TGG |  | 1123 | Fw |
| Y | amoA187f (amoA-1FF) | CAA TGG TGG CCG GTT GT |  | 1156 | Fw |
| Y | amoA310f (amoA-3F) | CGT GAG TGG GYT AAC MG |  | 1279 | Fw |
| Y – used in this study | amoA332f (amoA-1F) | GGG GTT TCT ACT GGT GGT |  | 1301 | Fw |
| n | amoA332fHY (amoA1F mod) | GGG GHT TYT ACT GGT GGT |  | 1301 | Fw |
| n | amoA337p (A337) | TTC TAC TGG TGG TCR CAC TAC CCC ATC AAC T |  | 1306 | Fw |
| n | amoA349r | ACC ACC AGT AGA AAC CCC |  | 1301 | Rev |
| n | amoA359rC (amoA-4R) | GGG TAG TGC GAC CAC CAG TA |  | 1309 | Rev |
| n | amoA627r | CGT ACC TTT TTC AAC CAT CC |  | 1577 | Rev |
| n | amoA664f | GCS TTC TTC TCN GCS TTTC |  | 1633 | Fw |
| n | amoA665r (AMO-R2) | GCT GCA ATA ACT GTG GTA |  | 1617 | Rev |
| n | amoA680r (A682 mod) | AAV GCV GAG AAG AAW GC |  | 1633 | Rev |
| n | amoA681r (A682) | GAA SGC NGA GAA GAA SGC |  | 1633 | Rev |
| n | amoA686r (AMO-R) | GAT ACG AAC GCA GAG AAG |  | 1638 | Rev |
| n | amoA802f | GAA GAA GGC TTT SCM GAG GGG |  | 1771 | Fw |
| Y | amoA820r (Amoa-2R′) | CCT CKG SAA AGC CTT CTT C |  | 1771 | Rev |
| Y – used in this study | amoA822r (amoA-2R) | CCC CTC KGS AAA GCC TTC TTC |  | 1771 | Rev |
| Y | amoA822rTC (amoA-2R-TC) | CCC CTC TGC AAA GCC TTC TTC |  | 1771 | Rev |
| Y | amoA822rTG (amoA-2R-TG) | CCC CTC TGG AAA GCC TTC TTC |  | 1771 | Rev |
| Y | amoA828r (302R) | TTT GAT CCC CTC TGG AAA GCC TTC TTC |  | 1771 | Rev |
| ***Archaea*** |  |  |  |  |  |
| Y – used in this study | Arch-amoAF | STA ATG GTC TGG CTT AGA CG |  | 1001 | Fw |
| Y | CrenAmo1F | AAT GGT CTG GCT WAG ACG C |  | 1003 | Fw |
| Y | Arch_amoA_F | AAT GGT CTG GST TAG AMG |  | 1003 | Fw |
| Y | crenAMO_F | ATG GTC TGG CTA AGA CGM TGT A |  | 1004 | Fw |
| Y | CrenamoA23f | ATG GTC TGG CTW AGA CG |  | 1004 | Fw |
| Y | CamoA-19f | ATG GTC TGG YTW AGA CG |  | 1004 | Fw |
| Y | CG I.1b-amoAF | ATA GTT GTA GTT GCT GTA AAT AG |  | 1040 | Fw |
| Y | CG I.1b-amoAF *real time* | GTA CAT TAT TGA CAA TCA ACG C |  | 1062 | Fw |
| Y | Arch-amoA-79F | ATT AAY GCA GGW GAY TAY A |  | 1076 | Fw |
| Y | Arch-amoA26F | GAC TAC ATM TTC TAY ACW GAY TGG GC |  | 1088 | Fw |
| Y – used in this study | AOA111F | TTY TAY ACH GAY TGG GCH TGG ACA TC |  | 1097 | Fw |
| n | amo196F | GGW GTK CCR GGR ACW GCM |  | 1193 | Fw |
| n | Arch-amoAF | GCT CTA AAT ATG ACA GTA TAC |  | 1220 | Fw |
| n | amo247 | CCA ACC AWG CWC CYT TKG CDA CCC |  | 1245 | Fw |
| n | CG I.1b-amoAR *real time* | ATC CTA RYG CAA ACC AAG CTC |  | 1257 | Rev |
| n | amo227R | CRA TGA AGT CRT AHG GRT ADC C |  | 1277 | Rev |
| n | Arch-amoAR | AYC ATG TTG AAY AAT GGT AAT GAC |  | 1405 | Rev |
| n | CG I.1b-amAR | CTC TAG AGG GTC TCT GAC CAG |  | 1439 | Rev |
| n | Arch-amoA-479R | TAT GGT GGY AAY GTD GGT C |  | 1479 | Rev |
| n | Arch-amoA417R | GGK GTC ATR TAT GGW GGY AAY GTT GG |  | 1481 | Rev |
| Y | CrenamoA616r | GCC ATC CAT CTG TAT GTC CA |  | 1616 | Rev |
| Y | CamoA-616r | GCC ATC CAB CKR TAN GTC CA |  | 1616 | Rev |
| Y – used in this study | Arch-amoAR | GCG GCC ATC CAT CTG TAT GT |  | 1619 | Rev |
| Y – used in this study | T7-AOA643R | TCC CAC TTW GAC CAR GCG GCC ATC CA |  | 1628 | Rev |
| Y | CrenAmo1R | GAC CAR GCG GCC ATC CA |  | 1628 | Rev |
| Y | cren AMO_F | CCC ACT TTG ACC AAG CGG CCA T |  | 1631 | Rev |

Supplementary Information 4: Applicability of alternative amoA PCR primer sets for use with the array

References:

1. Sinigalliano CD, Kuhn DN, Jones RD (1995) Amplification of the amoA gene from diverse species of ammonium-oxidizing bacteria and from an indigenous bacterial population from seawater. Appl Environ Microbiol 61: 2702-2706.

2. Molina V, Ulloa O, Farias L, Urrutia H, Ramirez S, et al. (2007) Ammonia-oxidizing beta-proteobacteria from the oxygen minimum zone off northern Chile. Appl Environ Microbiol 73: 3547-3555.

3. Juretschko S, Timmermann G, Schmid M, Schleifer KH, Pommerening-Roser A, et al. (1998) Combined molecular and conventional analyses of nitrifying bacterium diversity in activated sludge: Nitrosococcus mobilis and Nitrospira- like bacteria as dominant populations. Applied and Environmental Microbiology 64: 3042-3051.

4. Junier P, Kim OS, Molina V, Limburg P, Junier T, et al. (2008) Comparative in silico analysis of PCR primers suited for diagnostics and cloning of ammonia monooxygenase genes from ammonia-oxidizing bacteria. FEMS Microbiol Ecol 64: 141-152.

5. Norton JM, Alzerreca JJ, Suwa Y, Klotz MG (2002) Diversity of ammonia monooxygenase operon in autotrophic ammonia-oxidizing bacteria. Arch Microbiol 177: 139-149.

6. Webster G, Embley TM, Prosser JI (2002) Grassland management regimens reduce small-scale heterogeneity and species diversity of beta-proteobacterial ammonia pxidizer populations. Appl Environ Microbiol 68: 20-30.

7. Holmes AJ, Costello A, Lidstrom ME, Murrell JC (1995) Evidence that particulate methane monooxygenase and ammonia monooxygenase may be evolutionarily related. FEMS Microbiology Letters 132: 203-208.

8. Hoshino T, Noda N, Tsuneda S, Hirata A, Inamori Y (2001) Direct detection by in situ PCR of the amoA gene in biofilm resulting from a nitrogen removal process. ApplEnviron Microbiol 67: 5261-5266.

9. Purkhold U, Pommerening-Roser A, Juretschko S, Schmid MC, Koops HP, et al. (2000) Phylogeny of all recognized species of ammonia oxidizers based on comparative 16S rRNA and amoA sequence analysis: implications for molecular diversity surveys. Applied and Environmental Microbiology 66: 5368-5382.

10. Rotthauwe JH, Witzel KP, Liesack W (1997) The ammonia monooxygenase structural gene amoa as a functional marker: molecular fine-scale analysis of natural ammonia- oxidizing populations. Applied and Environmental Microbiology 63: 4704-4712.

11. Stephen JR, Chang YJ, Macnaughton SJ, Kowalchuk GA, Leung KT, et al. (1999) Effect of toxic metals on indigenous soil beta-subgroup proteobacterium ammonia oxidizer community structure and protection against toxicity by inoculated metal-resistant bacteria. Appl Environ Microbiol 65: 95-101.

12. Okano Y, Hristova KR, Leutenegger CM, Jackson LE, Denison RF, et al. (2004) Application of real-time PCR to study effects of ammonium on population size of ammonia-oxidizing bacteria in soil. Appl Environ Microbiol 70: 1008-1016.

13. Junier P, Kim OS, Junier T, Ahn TS, Imhoff JF, et al. (2009) Community analysis of betaproteobacterial ammonia-oxidizing bacteria using the amoCAB operon. Appl Microbiol Biotechnol 83: 175-188.

14. Nold SC, Zhou J, Devol AH, Tiedje JM (2000) Pacific Northwest marine sediments contain ammonia-oxidizing bacteria in the beta subdivision of the Proteobacteria. ApplEnviron Microbiol 66: 4532-4535.

15. Nicolaisen MH, Ramsing NB (2002) Denaturing gradient gel electrophoresis (DGGE) approaches to study the diversity of ammonia-oxidizing bacteria. J Microbiol Methods 50: 189-203.

16. Francis CA, Roberts KJ, Beman JM, Santoro AE, Oakley BB (2005) Ubiquity and diversity of ammonia-oxidizing archaea in water columns and sediments of the ocean. Proc Natl Acad Sci U S A 102: 14683-14688.

17. Konneke M, Bernhard AE, de la Torre JR, Walker CB, Waterbury JB, et al. (2005) Isolation of an autotrophic ammonia-oxidizing marine archaeon. Nature 437: 543-546.

18. de la Torre JR, Walker CB, Ingalls AE, Konneke M, Stahl DA (2008) Cultivation of a thermophilic ammonia oxidizing archaeon synthesizing crenarchaeol. Environ Microbiol 10: 810-818.

19. Hallam SJ, Konstantinidis KT, Putnam N, Schleper C, Watanabe Y, et al. (2006) Genomic analysis of the uncultivated marine crenarchaeote Cenarchaeum symbiosum. Proc Natl Acad Sci U S A 103: 18296-18301.

20. Tourna M, Freitag TE, Nicol GW, Prosser JI (2008) Growth, activity and temperature responses of ammonia-oxidizing archaea and bacteria in soil microcosms. Environ Microbiol 10: 1357-1364.

21. Pester M, Rattei T, Flechl S, Grongroft A, Richter A, et al. (2011) amoA-based consensus phylogeny of ammonia-oxidizing archaea and deep sequencing of amoA genes from soils of four different geographic regions. Environ Microbiol.

22. Park SJ, Park BJ, Rhee SK (2008) Comparative analysis of archaeal 16S rRNA and amoA genes to estimate the abundance and diversity of ammonia-oxidizing archaea in marine sediments. Extremophiles 12: 605-615.

23. Urakawa H, Tajima Y, Numata Y, Tsuneda S (2008) Low temperature decreases the phylogenetic diversity of ammonia-oxidizing archaea and bacteria in aquarium biofiltration systems. Appl Environ Microbiol 74: 894-900.

24. Treusch AH, Leininger S, Kletzin A, Schuster SC, Klenk HP, et al. (2005) Novel genes for nitrite reductase and Amo-related proteins indicate a role of uncultivated mesophilic crenarchaeota in nitrogen cycling. Environ Microbiol 7: 1985-1995.
