# Supplementary material for: High-Throughput Analysis of Ammonia Oxidiser Community Composition via a Novel, amoA-Based Functional Gene Array
Source: PLoS One. 2012 Dec 19;7(12):e51542. doi: 10.1371/journal.pone.0051542 (PMC3526613; doi:10.1371/journal.pone.0051542)
Supplement: Supporting Information S7 — Evaluation with environmental samples, full results. Nitrifier community analyses. Results of individual microarray experiments were first normalized to positive control probes, and then to the reference values determined individually for each probe (see Experimental procedures for details), averaged between replicate spots and displayed using the GeneSpring software. In essence, a value of 100% indicates maximum achievable signal for an individual probe, whereas a value of 10% indicates that about 10% of the total PCR product hybridised to that probe. Heat map colour coding is indicated on the side bar. Probes are indicated on the left side of the heat maps, by names and by numbers corresponding to Supporting Information S2. Major clusters are indicated by names and colours (see also Fig. 1; colours correspond between these two figures). A) AOA results; E1–E6: Temperate estuarine sediment samples (Derwent River, Tasmania). E1: upstream; E6: mouth. Os-Ccl: Open ocean water samples (Kimberley region, Western Australia). Os – Open ocean, surface; Ocl – Open ocean, chlorophyll maximum layer; Cs – Coastal area, surface; Ccl – Coastal area, chlorophyll maximum layer. B) AOB results; Sc, St, StN: Agricultural soil samples (Harden, New South Wales). Sc: control; St: tillage treated; StN: tillage treated with nitrogen amendment. W1–W3: Wastewater treatment plant water samples (Sydney, New South Wales). E1–E6: Temperate estuarine sediment samples (Derwent River, Tasmania). E1: upstream; E6: mouth. C) Side bar indicating heat map colour coding. Probe AamoA-159 has been discarded based on evaluation with estuarine sediment samples (see Results for details). The probe is therefore not listed in the final probe set (Supporting Information S1, Supporting Information S2, Supporting Information S5, Supporting Information S6), however is shown here to illustrate the process. (PDF) [file pone.0051542.s007.pdf]

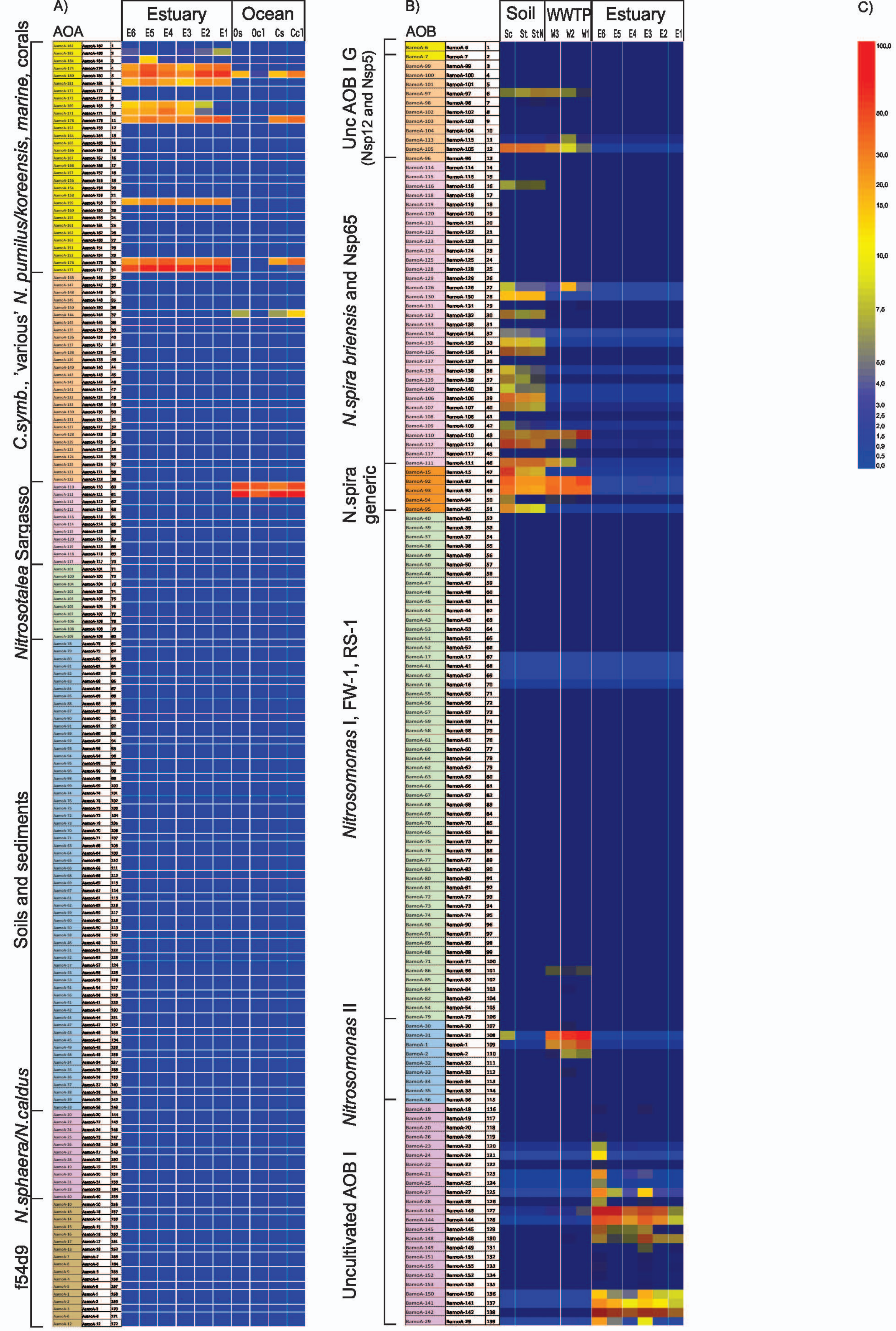

SI 7: Evaluation with environmental samples, full results

Nitrifier community analyses. Results of individual microarray experiments were first normalized to positive control probes, and then to the reference values determined individually for each probe (see Experimental procedures for details), averaged between replicate spots and displayed using the GeneSpring software. In essence, a value of 100% indicates maximum achievable signal for an individual probe, whereas a value of 10% indicates that about 10% of the total PCR product hybridised to that probe. Heat map colour coding is indicated on the side bar. Probes are indicated on the left side of the heat maps, by names and by numbers corresponding to SI 2. Major clusters are indicated by names and colours (see also Fig. 1; colours correspond between these two figures).

A) AOA results; E1-E6: Temperate estuarine sediment samples (Derwent River, Tasmania). E1: upstream; E6: mouth. Os-Ccl: Open ocean water samples (Kimberley region, Western Australia). Os – Open ocean, surface; Ocl – Open ocean, chlorophyll maximum layer; Cs – Coastal area, surface; Ccl – Coastal area, chlorophyll maximum layer.

B) AOB results; Sc, St, StN: Agricultural soil samples (Harden, New South Wales). Sc: control; St: tillage treated; StN: tillage treated with nitrogen amendment. W1-W3: Wastewater treatment plant water samples (Sydney, New South Wales). E1-E6: Temperate estuarine sediment samples (Derwent River, Tasmania). E1: upstream; E6: mouth.

C) Side bar indicating heat map colour coding.

Probe AamoA-159 has been discarded based on evaluation with estuarine sediment samples (see Results for details). The probe is therefore not listed in the final probe set (SI 1, SI 2, SI 5, SI 6), however is shown here to illustrate the process.
