# Supplementary material for: High-Throughput Analysis of Ammonia Oxidiser Community Composition via a Novel, amoA-Based Functional Gene Array
Source: PLoS One. 2012 Dec 19;7(12):e51542. doi: 10.1371/journal.pone.0051542 (PMC3526613; doi:10.1371/journal.pone.0051542)
Supplement: Supporting Information S8 — amoA array layouts and hybridisation examples. A. Schematic diagram of the microarray and slide design. Each slide contained three arrays (for three separate assays). Each array consisted of three replicate subarrays. Frames indicate universal probes spotted in multiple copies and spots with an external positive control probe (‘hyaBp’; results of this were not considered or used in the present study). B. Detailed design of a single array with exact positions for each probe. C. Representative hybridisation. Microarray image was adjusted for best viewing (quantitative conclusions drawn from the image may be misleading). (PDF) [file pone.0051542.s008.pdf]

A)

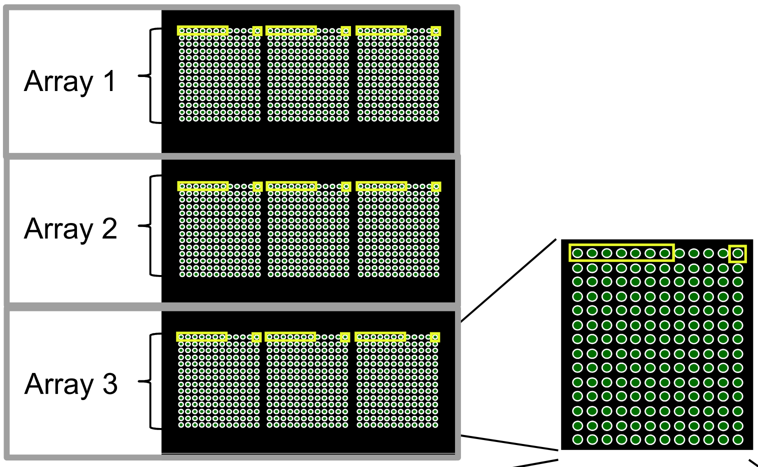

B)

hyaBp2	AOA111F	AOA643R-c	Arch-amoAF	Arch-amoAR-c	amoA-23F	amoA-616R-c	AamoA-181	AamoA-182	AamoA-183	AamoA-184	hyaBp2
AamoA-1	AamoA-2	AamoA-3	AamoA-4	AamoA-5	AamoA-6	AamoA-7	AamoA-8	AamoA-9	AamoA-10	AamoA-11	AamoA-12
AamoA-13	AamoA-14	AamoA-15	AamoA-16	AamoA-17	AamoA-18	AamoA-19	AamoA-20	AamoA-21	AamoA-22	AamoA-23	AamoA-24
AamoA-25	AamoA-26	AamoA-27	AamoA-28	AamoA-29	AamoA-30	AamoA-31	AamoA-32	AamoA-33	AamoA-34	AamoA-35	AamoA-36
AamoA-37	AamoA-38	AamoA-39	AamoA-40	AamoA-41	AamoA-42	AamoA-43	AamoA-44	AamoA-45	AamoA-46	AamoA-47	AamoA-48
AamoA-49	AamoA-50	AamoA-51	AamoA-52	AamoA-53	AamoA-54	AamoA-55	AamoA-56	AamoA-57	AamoA-58	AamoA-59	AamoA-60
AamoA-61	AamoA-62	AamoA-63	AamoA-64	AamoA-65	AamoA-66	AamoA-67	AamoA-68	AamoA-69	AamoA-70	AamoA-71	AamoA-72
AamoA-73	AamoA-74	AamoA-75	AamoA-76	AamoA-77	AamoA-78	AamoA-79	AamoA-80	AamoA-81	AamoA-82	AamoA-83	AamoA-84
AamoA-85	AamoA-86	AamoA-87	AamoA-88	AamoA-89	AamoA-90	AamoA-91	AamoA-92	AamoA-93	AamoA-94	AamoA-95	AamoA-96
AamoA-97	AamoA-98	AamoA-99	AamoA-100	AamoA-101	AamoA-102	AamoA-103	AamoA-104	AamoA-105	AamoA-106	AamoA-107	AamoA-108
AamoA-109	AamoA-110	AamoA-111	AamoA-112	AamoA-113	AamoA-114	AamoA-115	AamoA-116	AamoA-117	AamoA-118	AamoA-119	AamoA-120
AamoA-121	AamoA-122	AamoA-123	AamoA-124	AamoA-125	AamoA-126	AamoA-127	AamoA-128	AamoA-129	AamoA-130	AamoA-131	AamoA-132
AamoA-133	AamoA-134	AamoA-135	AamoA-136	AamoA-137	AamoA-138	AamoA-139	AamoA-140	AamoA-141	AamoA-142	AamoA-143	AamoA-144
AamoA-145	AamoA-146	AamoA-147	AamoA-148	AamoA-149	AamoA-150	AamoA-151	AamoA-152	AamoA-153	AamoA-154	AamoA-155	AamoA-156
AamoA-157	AamoA-158	AamoA-159	AamoA-160	AamoA-161	AamoA-162	AamoA-163	AamoA-164	AamoA-165	AamoA-166	AamoA-167	AamoA-168
AamoA-169	AamoA-170	AamoA-171	AamoA-172	AamoA-173	AamoA-174	AamoA-175	AamoA-176	AamoA-177	AamoA-178	AamoA-179	AamoA-180

C)

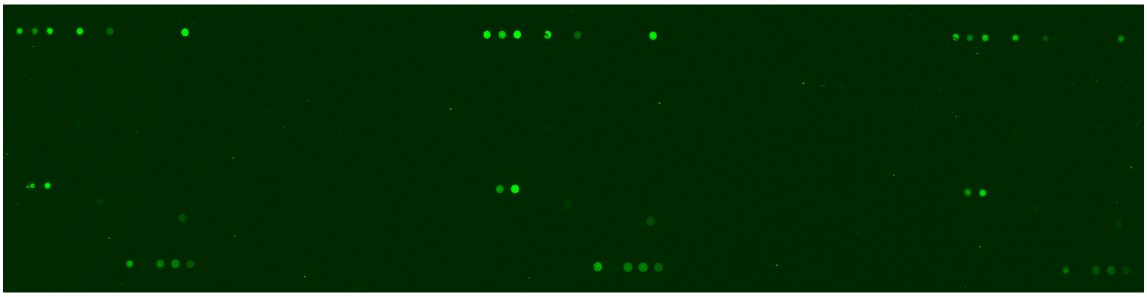

SI 8: *amoA* array layouts and hybridisation examples - AOA

A. Schematic diagram of the microarray and slide design. Each slide contained three arrays (for three separate assays). Each array consisted of three replicate subarrays. Frames indicate universal probes spotted in multiple copies and spots with an external positive control probe (‘hyaBp’; results of this were not considered or used in the present study).

B. Detailed design of a single array with exact positions for each probe.

C. Representative hybridisation. Microarray image was adjusted for best viewing (quantitative conclusions drawn from the image may be misleading).

A)

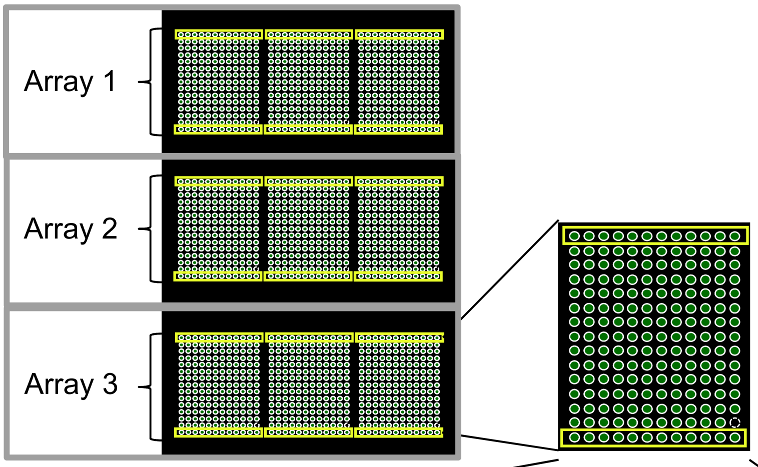

B)

hyaBp2	hyaBp2	amoA-1F	amoAf-i	amoA-2R-c	amoAr-i-c	mtrof173a	mtrof662-I	pmoA682-C	AMO-F	AMO-R-c	hyaBp2
BamoA-1	BamoA-2	BamoA-3	BamoA-4	BamoA-5	BamoA-6	BamoA-7	BamoA-8	BamoA-9	BamoA-10	BamoA-11	BamoA-12
BamoA-13	BamoA-14	BamoA-15	BamoA-16	BamoA-17	BamoA-18	BamoA-19	BamoA-20	BamoA-21	BamoA-22	BamoA-23	BamoA-24
BamoA-25	BamoA-26	BamoA-27	BamoA-28	BamoA-29	BamoA-30	BamoA-31	BamoA-32	BamoA-33	BamoA-34	BamoA-35	BamoA-36
BamoA-37	BamoA-38	BamoA-39	BamoA-40	BamoA-41	BamoA-42	BamoA-43	BamoA-44	BamoA-45	BamoA-46	BamoA-47	BamoA-48
BamoA-49	BamoA-50	BamoA-51	BamoA-52	BamoA-53	BamoA-54	BamoA-55	BamoA-56	BamoA-57	BamoA-58	BamoA-59	BamoA-60
BamoA-61	BamoA-62	BamoA-63	BamoA-64	BamoA-65	BamoA-66	BamoA-67	BamoA-68	BamoA-69	BamoA-70	BamoA-71	BamoA-72
BamoA-73	BamoA-74	BamoA-75	BamoA-76	BamoA-77	BamoA-78	BamoA-79	BamoA-80	BamoA-81	BamoA-82	BamoA-83	BamoA-84
BamoA-85	BamoA-86	BamoA-87	BamoA-88	BamoA-89	BamoA-90	BamoA-91	BamoA-92	BamoA-93	BamoA-94	BamoA-95	BamoA-96
BamoA-97	BamoA-98	BamoA-99	BamoA-100	BamoA-101	BamoA-102	BamoA-103	BamoA-104	BamoA-105	BamoA-106	BamoA-107	BamoA-108
BamoA-109	BamoA-110	BamoA-111	BamoA-112	BamoA-113	BamoA-114	BamoA-115	BamoA-116	BamoA-117	BamoA-118	BamoA-119	BamoA-120
BamoA-121	BamoA-122	BamoA-123	BamoA-124	BamoA-125	BamoA-126	BamoA-127	BamoA-128	BamoA-129	BamoA-130	BamoA-131	BamoA-132
BamoA-133	BamoA-134	BamoA-135	BamoA-136	BamoA-137	BamoA-138	BamoA-139	BamoA-140	BamoA-141	BamoA-142	BamoA-143	BamoA-144
BamoA-145	BamoA-146	BamoA-147	BamoA-148	BamoA-149	BamoA-150	BamoA-151	BamoA-152	BamoA-153	BamoA-154	BamoA-155	Blank
hyaBp2	hyaBp2	amoA-1F	amoAf-i	amoA-2R-c	amoAr-i-c	mtrof173a	mtrof662-I	pmoA682-C	AMO-F	AMO-R-c	hyaBp2

C)

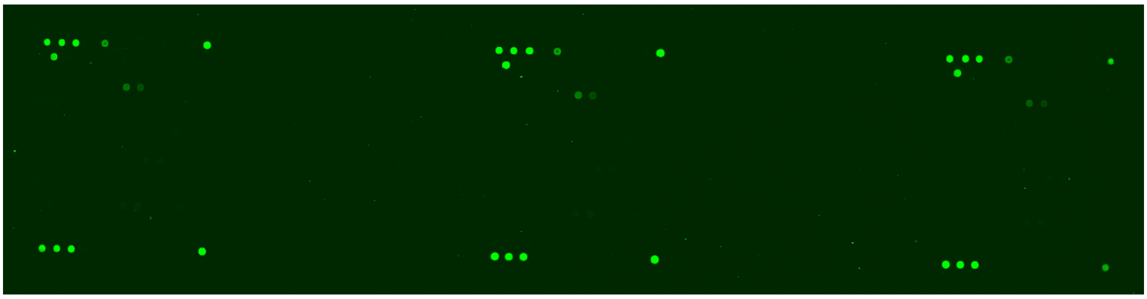

SI 8: *amoA* array layouts and hybridisation examples - AOB

A. Schematic diagram of the microarray and slide design. Each slide contained three arrays (for three separate assays). Each array consisted of three replicate subarrays. Frames indicate universal probes spotted in multiple copies and spots with an external positive control probe ('hyaBp'; results of this were not considered or used in the present study).

B. Detailed design of a single array with exact positions for each probe.

C. Representative hybridisation. Microarray image was adjusted for best viewing (quantitative conclusions drawn from the image may be misleading).
